# Testing the biodegradability of difficult compounds: a future challenge for the OECD/ISO standardization

**DOI:** 10.1007/s00253-026-13798-x

**Published:** 2026-03-24

**Authors:** Uwe Strotmann, Hermann J. Heipieper, Christian Eberlein, Philipp Mayer, Heidi Birch, Stefan Gartiser, Udo Pagga, Soumya Daturpalli, Glauco Battagliarin, Kathleen McDonough, Gerald Thouand

**Affiliations:** 1 Department of Chemistry, Westfälische Hochschule, Recklinghausen, Germany; 2 Department of Molecular Environmental Biotechnology, Helmholtz Centre for Environmental Research - UFZ, Leipzig, Germany; 3 Department of Environmental and Resource Engineering, Technical University of Denmark, Bygningstorvet, Building 115, 2800 Kgs., Lyngby, Denmark; 4 Hydrotox GmbH, Bötzinger Str. 29, 79111 Freiburg, Germany; 5 Ludwigshafen, Germany; 6 BASF SE, Carl-Bosch-Strasse 38, 67056 Ludwigshafen, Germany; 7 Procter and Gamble Company, 8700 S. Mason Montgomery Rd, Mason, OH 45040 USA; 8 Nantes University, ONIRIS, CNRS, GEPEA, UMR 6144, 85000 La Roche Sur Yon, France, France

**Keywords:** Chemicals, Biodegradation, OECD/ISO standardized tests, UVCBs, Hydrophobic compounds, Polymers, Microbial inocula

## Abstract

**Abstract:**

For a period exceeding five decades, industrial and scientific communities, in conjunction with regulators, have utilized a complexified, standardized system (e.g., OECD, Organisation for Economic Co-operation and Development; ISO, International Organization for Standardization; ASTM, American Society for Testing and Materials; CEN, Comité Européen de Normalisation) for the estimation of biodegradability of organic compounds. This system has been adopted in numerous countries worldwide and has also been integrated into European legislation (REACH, registration, authorisation, and restriction of chemicals). In recent years, a number of deficiencies have been identified in the standardized biodegradation test systems. This comprehensive review sets out the fields in which improvements are necessary to set up the next generation of reliable, standardized biodegradation tests. The main focus of the review is the challenges and modifications needed to test difficult-to-test compounds such as volatile, hydrophobic compounds, UVCBs (unknown or variable composition, complex reaction products or biological materials), water-soluble polymers, and plastics. Recent advances in the characterization of inocula for biodegradation tests are also addressed, which offer a valuable opportunity to enhance the reliability and reproducibility of biodegradation assays. Moreover, the potential for predicting biodegradation in the environment is a subject that is discussed in this text.

**Key points:**

*• It is essential that the OECD system of biodegradability tests be subjected to a thorough re-examination and further technical development.*

*• It is evident that UVCBs, hydrophobic compounds, and polymers present particular challenges in the context of OECD/ISO biodegradation tests.*

*• It is necessary that inocula for OECD/ISO-based biodegradation tests are characterized in a much more comprehensive manner.*

**Supplementary Information:**

The online version contains supplementary material available at 10.1007/s00253-026-13798-x.

## Introduction

Biodegradation research of organic compounds has been performed for several decades, and since the 1960 s, a large battery of standardized test methods has been developed by industrial, academic, and regulatory communities and published by ISO, OECD, CEN, and ASTM to name a few. Please refer to Table 1 for a list of common abbreviations used when describing the different test systems and methods.

**Table 1:** List of common abbreviations

Abbreviation	Detailed form
AFNOR	Association française de normalization
ASTM	American Society for Testing and Materials
BAP	Biodegradation adaptation potential
BOD	Biochemical oxygen demand
CEN	Comité Européen de Normalisation
CFU	Colony-forming units
CLPP	Community-Level Physiological Profiling
CMC	Carboxymethyl cellulose
COD	Chemical oxygen demand
CONCAWE	Conservation of Clean Air and Water in Europe
CRP	Chemical resistance potential
CS	Capsule suspension
DDT	Dichlorodiphenyltrichloroethane
DH	Degree of hydrolysis
DIN	Deutsches Institut für Normung (German Insitute for Normalization)
DOC	Dissolved organic carbon
DS	Degree of substitution
EC_50_	Effective concentration causing 50% inhibition
ECETOC	European Centre for Ecotoxicology and Toxicology of Chemicals
ECHA	European Chemicals Agency
EMPA	Eidgenössische Materialprüfungs- und Forschungsanstalt (Swiss federal laboratories for materials science and testing)
EN	European norm
EPF	Epifluorescence microscopy
eRBT	Enhanced ready biodegradability test
EU	European Union
F/M ratio	Food/biomass ratio
FCM	Fluorescence microscopy
GC/MS	Gas chromatography/mass spectrometry
GPC	Gel permeation chromatography
ISO	International organization for standardization
K_OW_	Octanol/Water partition coefficient
L-GLDA	Glutamate-*N,N*-diacetate
MITI	Ministry of international trade and industry (Japan)
MLSS	Mixed liquid suspended solids
MNP	Micro- and nanoplastics
MW	Molecular weight
OECD	Organisation for Economic Co-operation and Development
PAM	Polyacrylamide
PAsA	Polyaspartic acid
PBAT	Polybutyleneadipate-co-terephthalate
PBT	Persistence, bioaccumulation and toxicity
PCL	Polycaprolactone
PE	Polyethylene
PEG	Polyethylene glycol
PEO	Polyethylene oxide
PET	Polyethyleneterephthalate
PHA	Polyhydroxyalkanoate
PHBV	Poly(hydroxybutyrate)-co-(3-hydroxyvalerate)
PP	Polypropylene
PPI	Physiological potential of an inoculum
PS	Polystyrene
PUA	Polyurea
PVA	Polyvinyl alcohol
PVC	Polyvinylchloride
QSAR	Quantitative structure activity relationship
RBT	Ready biodegradability test
REACH	Registration, authorisation, and restriction of chemicals
SCAS test	Semi-continuous activated sludge test
SDA	Soap and detergents association
SS	Suspended solids
TC	Technical committee
TG	Technical guideline
ThOD	Theoretical oxygen demand
TOC	Total organic carbon
UVCB	Substances of unknown or variable composition, complex reaction products or biological materials
VSS	Volatile suspended solids
WSP	Water soluble polymer
WWTP	Wastewater treatment plant

The main aim of these tests is to evaluate the biodegradability of organic compounds utilizing inocula from different environmental compartments. However, some of the tests are also used to assess chemical persistence. The tiered test system created by the OECD is of particular significance and has been adopted on a global scale. The system has exerted a profound influence on legislation in European countries through the European Union’s REACH legislation, which was designed to protect human health and the environment from chemical risks. The OECD guidelines are organized in a three-step-tiered test sequence where the tests on ready biodegradability (OECD 301 1992) are usually the first step and are used to evaluate the ready biodegradability and thus non-persistence of chemicals under nearly all natural conditions. The second tier contains the tests for inherent biodegradability (OECD 302A 1981; OECD 302B 1992; OECD 302C 1981). The highest tier consists of simulation tests (OECD 303A 2001; OECD 307 2002; OECD 308 2002; OECD 309 2004; OECD 314 2008) which are used to assess biodegradability under more realistic environmental conditions, for example in biological wastewater treatment plants (WWTPs). Additionally, simulation tests are used for determining the half-lives and rates of primary and ultimate biodegradation of chemicals in key environmental compartments for use in quantitative environmental risk assessments (European Chemicals Agency (ECHA 2016)). Results of these assays are also used to estimate chemical persistence (e.g., half-lives > 60 days in marine water and half-lives > 40 days in freshwater or estuarine water) (ECHA). Details of the EU-tiered test system to evaluate persistence have been reviewed by Kowalczyk et al. (2015) and Strotmann et al. (2023). An overview of the system, along with its close connection to the ISO system, is presented in Table 2.

**Table 2:** Important OECD-based biodegradation tests, their history, and connection to the ISO system. AFNOR, Association française de normalization; MITI, Ministry of International Trade and Industry; SDA, Soap and Detergent Association

OECD guideline	Test	First most relevant reference	Current version	Corresponding ISO norm
**A. Ready biodegradability tests (screening tests)**				
301 A	DOC-die away test	AFNOR (1977)	1992	ISO 7827 (2010)
301 B	CO_2_ evolution test	Sturm (1973)	1992	ISO 9439 (1999)
301 C	MITI-(I); respirometric test based on oxygen consumption	MITI (1978b)	1992	–
301 D	Closed bottle test	Fischer (1963)	1992	ISO 10707 (1994)
301 E	Modified OECD screening test (based on earlier screening tests for the determination of the primary degradation of surfactants: shake flask tests of SDA)	Heinz and Fischer (1964); Struijs and Stoltenkamp (1994)	1992	ISO 7827 (2010)
301 F	Manometric respirometry test	Painter and King (1985)	1992	ISO 9408 (1999)
310	CO_2_ headspace test	Gledhill (1975)	2014	ISO 14593 (1999)
**B. Inherent biodegradability tests (screening tests)**				
302 A	Modified SCAS test (semi-continuous activated sludge test)	Soap and Detergent Association (1965)	1981	ISO 9887 (1992)
302 B	Zahn-Wellens/EMPA test	Zahn and Wellens (1974)	1992	ISO 9888 (1999)
302 C	Modified MITI (II) test	MITI (1978a) Biodegradability and bioaccumulation test of chemical organic compounds	1981	–
304 A	Inherent biodegradability in soil	Bartha and Pramer (1965)	1981	–
**C. Simulation tests (Screening tests)**				
303 A, B	Aerobic sewage treatment A: activated sludge units B: biofilms	Gerike and Fischer (1979; 1981); Painter and Bealing (1989)	2001	ISO 11733 (2004)
307	Aerobic and anaerobic transformation in soil	Guth (1981)	2002	–
308	Aerobic and anaerobic transformation in aquatic sediment systems	OECD (1995)	2002	–
309	Aerobic mineralization in surface water	Ingerslev and Nyholm (2000)	2004	ISO 14592–1 (2002); ISO 14592–2 (2002)
314 A to E	Simulation tests to assess the biodegradability of chemicals discharged in waste water OECD 314 A: Biodegradation in a sewer system test OECD 314 B: Biodegradation in activated sludge test OECD 314 C: Biodegradation in anaerobic digester sludge test OECD 314 D: Biodegradation in treated effluent-surface water mixing zone test OECD 314 E: Biodegradation in untreated wastewater-surface water mixing zone test	Federle and Itrich (1997); Itrich and Federle (2004); Matthijs et al. (1995); Nuck and Federle (1996); Steber and Wierich (1987)	2008	–
**D. Other special biodegradability tests**				
306	Biodegradability in seawater	Nyholm et al. (1992)	2001	ISO 16221 (2001)
311	Anaerobic biodegradability	Birch et al. (1989), Pagga and Beimborn 1993	2006	ISO 11734 (1995)

The significance of biodegradation testing cannot be overstated, and the methods employed are subject to ongoing scrutiny. The growth in knowledge, new technical possibilities, and innovations in efficient data analysis have led to ongoing discussion of technical deficiencies, the boundaries of existing tests, and the need for new test systems. As an example, when conducting degradation tests, it is necessary to exercise discernment in determining the discriminative limits between “ready” and “inherent” biodegradability of chemicals, as delineated by the OECD guidelines.

In contrast, the overarching issue has consistently been whether these test systems enable a reliable prediction of the behavior of the tested chemicals in different environmental compartments and for subsequent risk assessments. There are a number of arguments both for and against these tests, and a re-examination and further technical development of the entire test system are argued to be necessary (Davenport et al. 2022; Gartiser et al. 2007, 2017, 2022, 2023; Kaiser 1998; Kowalczyk et al. 2015; Pagga 1997; Painter 1995; Reuschenbach et al. 2003; Strotmann et al. 2023; Vazquez-Rodriguez and Beltran-Hernandez 2004).

Over the years, a significant number of chemicals have been thoroughly tested and regulated. However, further research on methods is particularly urgent for four classes of organic compounds. These include (1) volatile compounds, (2) hydrophobic compounds, (3) UVCBs (unknown or variable composition, complex reaction products, or biological materials), and (4) polymers (water-soluble polymers and plastics, including microplastics). Chemicals belonging to one of these four groups are often designated as difficult-to-test organic compounds with respect to biodegradability testing. These complex compounds therefore pose the next challenges in the field of biodegradability testing.

The question of how the next generation of standardized biodegradation tests should be designed to meet future demands is one that must be addressed. The present review commences with a concise historical account of the evolution of the complex system of standardized biodegradation tests. The primary focus of this study is on the various categories of challenging test compounds in biodegradation tests, the methods for accurately describing a reliable inoculum, and the possibilities of predicting biodegradation in the environment.

## A short history of standardized biodegradation tests: from ancient to present

### Terms and definitions

Key terms in biodegradation testing are summarized in Table 3. Biodegradation can be performed aerobically, whereby carbon sources are degraded to produce carbon dioxide, water, and some mineral salts. In anaerobic biodegradation, organic compounds are degraded to produce a mixture of methane and carbon dioxide (biogas), as well as water and mineral salts. Bacteria also use the breakdown of organic compounds to generate energy and form new biomass. Primary biodegradation is the first stage of biodegradation, meaning that an organic compound is not fully degraded but rather its structure is altered so that it loses its characteristic properties. Ultimate biodegradation is defined as the complete degradation of an organic compound.

**Table 3:** Glossary of important definitions concerning biodegradation

Specific term	Definition	References
Biodegradation	Aerobic or anaerobic degradation of organic chemical compounds by microorganisms	Alexander (1994)
Primary biodegradation	First stage of biodegradation, in which microorganisms alter the chemical structure of a substance	Painter (1995) OECD (2006)
Ultimate biodegradation	Complete biodegradation by microorganisms into basic elements like carbon dioxide, water, and mineral salts (mineralization)	Painter (1995) OECD (2006)
Ready biodegradability	An intrinsic compound property that ensures rapid and complete aerobic ultimate biodegradation under stringent test conditions	Painter (1995) OECD (2006)
Inherent biodegradability	An intrinsic compound property that ensures aerobic ultimate biodegradation while requiring favorable conditions or extended time	Painter (1995) OECD (2006)
Biodegradation simulation test	Test system that is used to investigate the biodegradation of a chemical substance under realistic environmental conditions	Painter (1995) OECD (2006)
Co-metabolism	Metabolic conversion of a specific organic compound during growth on another organic compounds	Alexander (1994) Painter (1995)
DOC die away test	Biodegradation test where the removal of dissolved organic carbon (DOC) is determined	Painter (1995) OECD (2006)
Respirometric test	Biodegradation test where the depletion of oxygen is determined	Painter (1995) OECD (2006)
CO_2_ evolution test	Biodegradation test where the production of carbon dioxide (CO_2_) is determined	Painter (1995) OECD (2006)
Multicomponent test	Biodegradation test where simultaneously the removal of DOC, the depletion of oxygen and the production of carbon dioxide are determined	Strotmann et al. (2004)

The initial biodegradation tests were conducted as aerobic tests in shake flasks or straightforward stirred and aerated reactors, in which the removal of dissolved organic carbon (DOC) was meticulously monitored. The inoculum was often activated sludge bacteria from a wastewater treatment plant or natural water bodies (e.g., lakes or rivers). These DOC-based tests are known as DOC die-away tests. Later, the production of carbon dioxide or use of oxygen was also measured, leading to CO₂ evolution tests and respirometric tests. The most reliable results are obtained using multicomponent test systems that combine DOC removal, oxygen consumption, and CO₂ production.

### The timeline of standardized biodegradation tests

The development of standardized biodegradation tests is closely connected to two different entrance gates of chemicals to the environment: the water compartment and the soil compartment. The focus on the water compartment commenced in the late 1950 s and 1960 s, where biodegradability tests were developed due to foaming detergents in wastewater treatment plants and rivers. A historic review about these early developments (Heinz and Fischer 1964) covers the development of different aerobic test systems ranging from river water die-away tests, shake flask tests, stirring tests, and bubble columns up to laboratory sewage treatment plants. The first biodegradation tests targeted primary biodegradation. Later, these tests were modified to also evaluate ultimate biodegradability not only of detergents but also of a wide range of other organic compounds. These developments were done by chemical companies and academic institutions throughout Europe, the USA, and Japan.

**Fig. 1:**
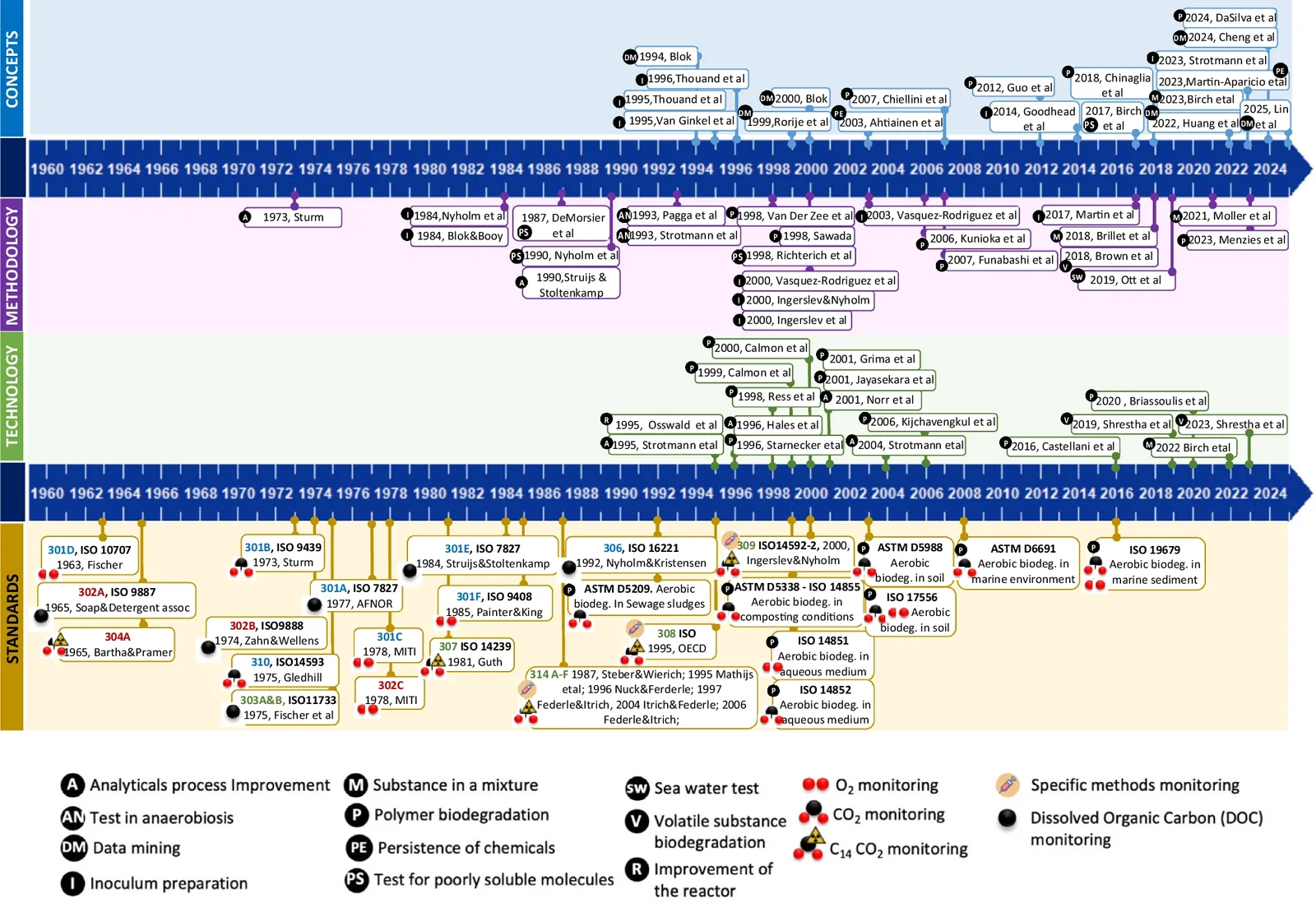
Timeline of the major developments in OECD/ISO-based biodegradation tests. The detailed references and ISO and ASTM standards for this figure can be found in the supplementary material S1. Main articles and standards from 1988 to 2024 covering four main domains on the topic of biodegradation evaluation either for soluble and poorly soluble substances and polymers. Method: Five hundred ten articles were selected from the SCOPUS data bank from 1988 to 2024 using the keywords Biodeg* and (test* or assay*) and applying the following journal filter categories: environment, polymers, chemistry, engineering, environmental engineering, water research, toxicology, microbial environments, polymer sciences, water resources, biotechnology, applied microbiology, and microbiology. This first set was screened, and articles out of the topics were withdrawn leading to a final set of 302 articles. From this last set, the most relevant papers were selected and organized in four main domains

The focus on the soil compartment was driven by the environmental concerns associated with insecticides such as dichlorodiphenyltrichloroethane (DDT), which were extensively used in plant treatments and found in soil. Concerns over the toxicity, persistence, and spread of these compounds have led to increased research into the biodegradation of pesticides (e.g., insecticides, herbicides, and fungicides) in the soil compartment (Colborn and Smolen 1996). The developments in the field of biodegradation have resulted in a wide range of regulatory needs, which have in turn led to the creation of a variety of test methods. A historical overview of the developments is given in Fig. 1.

To ensure a certain level of reproducibility and quality, standardized tests were developed by ISO, the OECD, and the ASTM. The OECD developed a three-tiered system that is widely accepted and comprises the following steps: (1) tests for ready biodegradability, (2) tests for inherent biodegradability, and (3) simulation tests. In 1981, OECD published the first OECD 301 A-F test guidelines for determining ready biodegradability, while adopting some national test guidelines (AFNOR, MITI) and adopting some test conditions such as the test duration and temperature. In the OECD system, the tests for ready and inherent biodegradability are regarded as biodegradation screening tests with a focus on intrinsic biodegradability of organic compounds. The screening tests are performed at unnaturally high test substance concentrations thus leading to biomass growth and biodegradation growth kinetics. This group of screening tests is completed by tests on biodegradability in seawater (OECD 306, 1992) and biodegradability under anaerobic conditions (OECD 311, 2006). The OECD simulation tests are performed at relatively low substance concentrations similar to those in the environment. Their focus is on the determination of biodegradation kinetics and possible transformation products.

In ready biodegradability and other screening tests, complications arose when poorly water soluble, volatile, or adsorbing compounds were tested. For this reason, a broadly diversified suite of test systems ranging from DOC removal tests and closed bottle tests to respirometric test systems and CO_2_ headspace test systems were established. This development took place from the 1970 s to the 1990s. Here, the newest developed test systems were often first standardized by ISO and later adopted by the OECD. The OECD 306 test was also elevated to the status of a “marine biodegradation screening test” following a prolonged period of development (Kowalczyk et al. 2015; Nyholm et al. 1992). The reason for this was that it was the only test that used seawater as inoculum.

The tiered OECD system forms the basis for chemical regulation in Europe, especially for the REACH persistence assessment framework where substances can be classified as not persistent (Not P), potentially persistent, persistent (P), and very persistent (vP) (Hughes et al. 2022a, 2022b). The different test systems and their underlying design have been summarized in various reviews (Pagga 1997; Painter 1995; Strotmann et al. 2023; Kowalczyk et al. 2015), including their respective advantages and disadvantages. In the context of risk assessment of organic compounds, certain aspects of the design of ready biodegradability tests have been discussed, including the food/biomass (F/M) ratio in the tests, the selection of appropriate reference compounds, the choice of inoculum, and the use of unnaturally high test substance concentrations. Furthermore, the ready biodegradability tests showed a high variation in test results and were often criticized for delivering false negative results (Kowalczyk et al. 2015; Painter 1995; Strotmann et al. 2023).

As illustrated in Fig. 1, the standardization of new test systems stagnated around 2000, and from then on, new developments were mainly published in scientific literature. An example of a later development that has not yet been adopted in the standards is the enhanced ready biodegradability test (eRBT) that extends the duration of a ready biodegradability test. It was developed since the duration of tests (for example, 28 days for OECD 301 tests) was decided based on pragmatic factors rather than scientific rationale. The eRBT can be utilized in the persistency assessment as weight of evidence in line with ready and inherent biodegradability tests (ECHA 2023a; Gartiser et al. 2023).

Another recent development is the combination tests and multicomponent test systems that enhance the accuracy of test results. These systems facilitate the measurement of multiple parameters, including DOC removal, oxygen depletion, and carbon dioxide formation, in a parallel manner. A methodology was formulated to estimate the heterotrophic yield coefficient of the degrading bacteria, thus facilitating a more profound comprehension of the biodegradation processes and potentially enabling the development of a significant next generation of test systems (Czechowska et al. 2013). However, these contemporary methodologies have not been incorporated into the prevailing OECD framework.

The testing of “difficult test substances” in terms of biodegradation has become a significant challenge from technical, scientific, and regulatory perspectives. Dedicated method modifications are often needed to accommodate and properly test such difficult substances. However, these method modifications are often viewed as deviations from standard test methods, which can result in the rejection of test results by the national competent authorities and confusion over which assays are applicable for different regulatory schemes. The situation is fundamentally different for the aquatic toxicity testing of difficult substances, where an OECD guidance document is in place for the guidance and the justification of test method modifications that are needed for the Aquatic Toxicity Testing of Difficult Test Chemicals (OECD 2019).

Lastly, there is a discussion about the environmental relevance of ready biodegradability tests. Conclusions regarding the degradation times of chemicals in the environment cannot be drawn from current RBTs operated under standardized conditions. This leads to a discrepancy between RBTs and simulation tests. Consequently, there is a demand for improved RBTs, as articulated by Boethling et al. (2009) and Brillet et al. (2016). It has been claimed that the predictive value of RBTs is improved by potential pre-adaptation processes (Thouand et al. 1996). An example is the CONCAWE (Conservation of Clean Air and Water in Europe) test, which aims to determine the biodegradability of oil products. This test was developed as an inherent OECD 310 biodegradation test (CO_2_ headspace test), allowing a preadapted inoculum. However, it was not accepted by the OECD (Battersby et al. 1999), since at present, OECD or ECHA guidance for assessing ready biodegradability or persistency does not permit pre-adaptation of the inoculum to the test substance. However, such tests have the potential to inform the further development of improved biodegradation assays.

In conclusion, it can be posited that contemporary techniques, including pre-adaptation of an inoculum, an extended test duration, and multicomponent test systems, represent a substantial enhancement to the existing repertoire of techniques. This, in turn, has the potential to propel the design of OECD test systems into the future. However, it would appear that further development is being stymied, at least in part, as a consequence of an overreliance on methodologies that have been approved by the relevant regulatory authorities.

## Biodegradability of hydrophobic compounds, volatile compounds, and UVCBs

Biodegradation testing of hydrophobic and volatile chemicals is important since hydrophobic chemicals are prone to bioaccumulation and semi-volatile chemicals are prone to long-range transport in the environment—properties of concern if chemicals are also persistent. The test challenges for hydrophobic and volatile compounds have been known for decades and are addressed in standardized guidelines (e.g., low water solubility is addressed in Annex III of OECD 301 (1992) and ISO 10634 (2018)). However, substantial test substance losses have been seen in standardized tests recommended for hydrophobic and volatile chemicals (Brown et al. 2018), and there is thus a need to revisit the guidance for these chemicals.

UVCB substances are multicomponent mixtures that often cannot be identified with reasonable certainty by their chemical composition because of their varying constituents, and they are therefore usually characterized by a combination of the source and processing (e.g., plant extract). Many UVCBs contain hydrophobic and volatile constituents (e.g., botanical oils and petroleum products). About 20 to 40% of the number of all registered chemicals in the USA and Europe are estimated to be UVCBs, which clearly indicates the technical and economic importance of this group (Lai et al. 2022). Although guidance has been published on testing UVCBs (ECHA Guidance R7b, ECHA, 2023c), methods for assessing the persistence of UVCBs via biodegradation tests are still at a method development stage (Birch et al. 2024; Davenport et al. 2022; Lai et al. 2022), and further guidance is therefore needed.

### Hydrophobic compounds

Hydrophobic chemicals are chemicals with low solubility and are generally considered to be chemicals with log octanol-water partition ratios (log *K*_ow_) above 3–4. The challenges in biodegradation testing of hydrophobic chemicals relate to their low water solubility, the increased risk of inhibition of biomass by these chemicals, and the potential for sorptive losses.

**Fig. 2:**
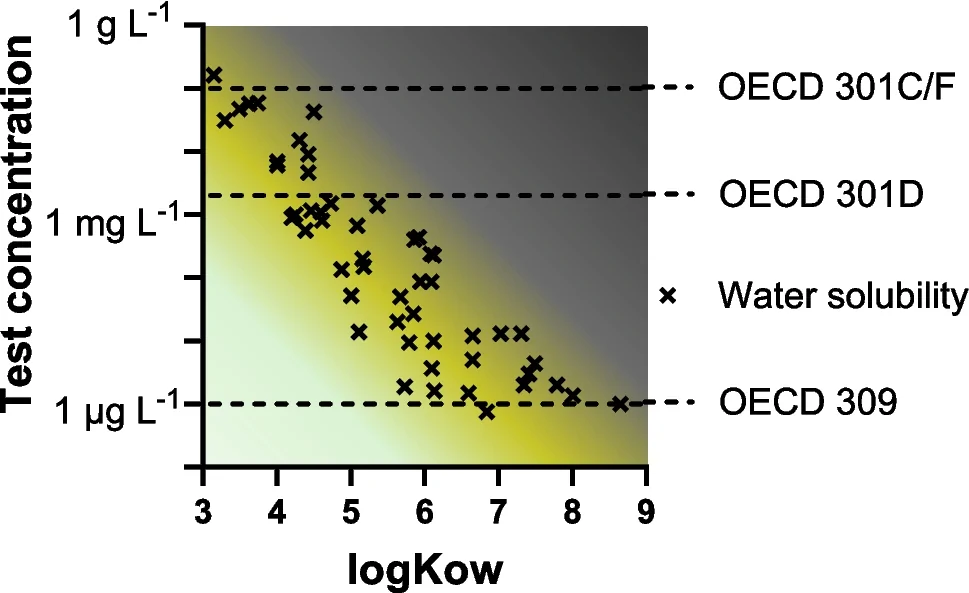
Test concentration relative to hydrophobicity. Water solubility of example chemicals: aliphatic and aromatic petroleum hydrocarbons (C_8_–C_20_) from Birch et al. (2018). The light green area (left bottom) indicates test concentrations where precipitation is not expected; the dark area indicates test concentrations where free phase chemical is more likely and dependent on sorption to inoculum constituents

In ready biodegradability tests (OECD 301, 1992), the testing of chemicals above their solubility is allowed (see Annex III of OECD 301). Figure 2 illustrates the relationship between hydrophobicity and the water solubility of example hydrocarbons (petroleum hydrocarbons covering aliphatic and aromatic compounds from size C_8_–C_20_) and shows that chemicals with log *K*_ow_ above 3–4 are often not fully dissolved at test concentrations used in OECD 301C/F and OECD 301D tests.

ISO 10634 (2018) provides recommendations on dosing procedures for poorly water-soluble compounds in biodegradation tests. The techniques include addition with inert support (e.g., silica gel), preparing dispersions of test chemical (e.g., ultrasonication), and preparing emulsions of test chemical (using, e.g., polymers or silicone oil). These methods change the partitioning processes in the test. Improved contact between the free phase chemical and microorganisms reduces the risk that bioavailability is rate limiting for biodegradation (either by improving dissolution kinetics or by growth directly on free phase chemical). However, if too much inert support or emulsifier is added, bioavailability and thus biodegradation rates could be reduced (ISO 10634 2018) because an extra sorbing phase is added to the test. Furthermore, it has been shown that close to the water solubility of chemicals, there is an increased risk of microbial inhibition due to baseline toxicity (Hammershøj et al. 2020; Schmidt and Mayer 2015). Reducing bioavailability may therefore reduce toxicity and thereby increase biodegradation in the test. The partitioning processes in a test system dosed above solubility and their effect on biodegradation are thus complicated and depend on the specific test chemical (Sweetlove et al. 2016; ISO 10634, 2018). Currently, no ready biodegradability tests exist that can accommodate poorly soluble chemicals below their water solubility because a certain concentration (e.g., 2 mg L^−1^) is needed to distinguish mineralization from inoculum respiration. The authors see this as a methodological gap that should be addressed by future scientific developments.

The OECD 309 simulation biodegradation test specifically states that chemicals should not be tested above their water solubility. Figure 2 illustrates that some hydrophobic chemicals may need lower test concentrations than generally used, which could conflict with detection limits for the analyses. The test concentration in soil and sediment tests may be higher than the water solubility of the test chemical because of sorption to soil or sediment; however, the freely dissolved concentrations can of course not be above water solubility.

Another challenge for hydrophobic chemicals is sorptive losses (e.g., absorption on plastic or rubber parts in a test system) that could lead to erroneous biodegradation results. The basic principle of a biodegradation test is violated if the test chemical is lost or not in contact with the microbial inoculum during the test. Sorptive losses can be minimized by avoiding plastic, rubber or other sorptive materials in contact with the test chemicals. For chemicals that are hydrophobic and non-volatile, this entails mainly surfaces in contact with the test solutions; however, for chemicals that are hydrophobic and slightly volatile, these materials should also not be in contact with the headspace.

While sorptive losses can be minimized, it may not be possible to completely avoid such losses. Sterile controls can then be used to account for minor abiotic losses in simulation biodegradation tests (OECD 309) (Birch et al. 2023). In the OECD 309 test, autoclaving, addition of sodium azide, mercury chloride, or formalin, or gamma irradiation is mentioned (OECD 309). Given the restrictions on mercury in the EU, mercuric chloride should not be used, and for work environment reasons and waste minimization, the handling of highly toxic chemicals should generally be minimized. Autoclaving can change the sorptive properties of sediment and soil, and gamma irradiation is not readily available in most routine labs. Ultrapure water can be used for abiotic controls in pelagic tests having low suspended solids and dissolved organic matter, but not if sediment or sludge is used in the test (Birch et al. 2023). There is thus not one optimal way to prepare sterile controls that is efficient and practical for all chemicals and test methods.

### Volatile compounds

The term “volatility” refers to two chemical properties, namely vapor pressure and Henry’s law constant. While the vapor pressure describes the equilibrium partitioning from a pure phase chemical (solid or liquid) to air, the Henry’s law constant describes its equilibrium partitioning between water and air. Ready biodegradability tests, inherent biodegradability tests, and most simulation biodegradation tests are aqueous tests (except soil tests). It is therefore generally appropriate to use Henry’s law constants to describe the volatility of chemicals in biodegradation tests.

The challenges for volatile chemicals in biodegradation tests are mainly evaporative losses during test setup, incubation, and sample preparation for analysis. For highly volatile chemicals, partitioning to the headspace of a test separates the test chemical from the microbial community in the water, sediment, or soil, and thus decreases the availability of the test chemical for the microbes (Birch et al. 2017; Shrestha et al. 2019, 2020). The partitioning to the headspace generally has fast equilibrium kinetics in test systems that are shaken or stirred, and unless the chemical is removed from the system into traps or by sorption to plastic, the chemical will re-enter the water phase as degradation occurs because the partition equilibrium shifts (e.g. Shrestha et al. 2020). If biodegradation depends on substrate concentration (e.g., first order kinetics), the lower aqueous concentration of the test chemical caused by volatilization will directly influence biodegradation kinetics (Birch et al. 2017). If the chemical is lost before, during, or after the test, the losses can lead to erroneous results and prevent the establishment of complete mass balances.

OECD 301 describes three closed tests (OECD 301C 1992; OECD 301D 1992; OECD 301F 1992) and is complemented by the closed test in OECD 310 (2014). In certain cases, test substances are volatile but not lipophilic. Then either of the closed tests can be used, bearing in mind that the larger headspace, the more likely that biodegradation will be slightly retarded because of lack of substance bioavailability. According to OECD 310 (2014), test substances up to Henry constants of 50 Pa m^3^ mol^−1^ can be tested based on the assumption that < 1% are present in the headspace when the headspace to liquid ratio is 1:2. In most cases, however, volatile chemicals are also lipophilic and will sorb to plastic and rubber if present. OECD 301D (1992) is the only OECD ready biodegradability test designed without a headspace and without plastic and rubber components that could trap hydrophobic volatile chemicals (plastic components in OECD 301C (1992) and F (1992) and rubber septa in OECD 310 (2014)). Brown et al. (2018) demonstrated how the standard manometric (OECD 301F 1992) test is not appropriate for volatile chemicals that are also lipophilic. However, the use of WWTP effluent as inoculum instead of activated sludge in the OECD 301D (1992) test, and the resulting increased test substance to biomass ratio (F/M ratio, see Table 4), leaves this test substantially different from the other OECD 301 test versions and the OECD 310 (2014). Brown et al. (2018) therefore designed a test that is a mix of the OECD 301D (1992) and OECD 301F (1992) tests, which uses activated sludge as inoculum and includes a headspace but avoids sorptive surfaces and uses contactless oxygen sensor spots to measure oxygen without opening the bottles. This test, or another format using the same test principle (Birch et al. 2025), is currently seen by the authors as a technically robust development for ready biodegradability tests of volatile and hydrophobic chemicals.

**Table 4:** Substrate and biomass concentrations of the different OECD screening test systems and of a real WWTP

Test system	DOC conc. of the test substance (mg L^−1^)	Test substance concentration (mg L^−1^)	Inoculum concentration (g MLSS L^−1^)	Initial F/M ratio (g DOC g^−1^ MLSS)
OECD 301 A (DOC die-away test)	*10–40*	A: 13–52 G: 25–100	*0.03*	0.33–1.33
OECD 301 B (CO_2_ evolution test)	*10–20*	A: 13–26 G: 25–50	*0.03*	0.33–0.67
OECD 301 C (MITI(I) test)	A:77 G: 40	*100*	*0.03*	A: 2.56** G: 1.33**
OECD 301 D (closed bottle test)	A: 1.5–7.7 G: 0.8–4.0	*2–10*	*9.2* × *10*^−5^***	A: 16.4–83.7** G: 8.7–43.5**
OECD 301 E (modified OECD screening test)	*10–40*	A: 13–52 G: 25–100	*9.2* × *10*^−6^***	1087–4348
OECD 301 F (manometric respirometry test)	A: 77 G: 40	*100*	*0.03*	A: 2.56** G: 1.33**
OECD 310 (CO_2_ headspace test)	*2–40*	A: 2.6–52 G: 5–100	*0.03*	0.067–1.33
OECD 302 A (modified SCAS test)	A: 15.4 G: 8	*20*	*1.0–4.0*	A: 0.0039–0.0154** G: 0.002–0.008**
OECD 302 B (Zahn-Wellens/EMPA test)	*50–400*	A: 65–519 G: 125–1000	*0.2–1.0*	0.25–2.00
OECD 302 C (modified MITI test II)	A: 0.023 G: 0.012	*0.03*	*1* × *10*^*−4*^	A: 0.23** G: 0.12**
Municipal WWTP (DOC data from Escalas et al. (2003); Tchobanoglous et al. (1991)	80–250	NA*	3.05	0.026 −0.082

*NA not applicable as this is not a test but instead provides average data from domestic WWTPs for DOC levels and MLSS for comparison purposes

**F/M ratio calculations were done for glucose (40% C content) and aniline (77% C content) as example compounds. The OECD screening study guidelines recommend aniline as a reference compound. Also, the resulting initial F/M ratio is indicated. A, aniline; G, glucose. OECD recommendations are indicated in italics. Calculations were performed with a total cell concentration of 3.3 × 10^12^ cells L^−1^ for activated sludge and 2 × 10^9^ cells L^−1^ for effluent (Foladori et al. 2010). The calculations were based on the assumption that the activated sludge concentration in the aeration basin of a wastewater treatment plant (WWTP) was 3.05 g MLSS (mixed liquid suspended solids) L^−1^, which is within the normal range for a municipal WWTP (Foladori et al. 2010). Therefore, it was assumed that one cell represents an MLSS of 9.2 × 10^−13^ g

***Inocula concentrations (g MLSS L^−1^) were calculated from approximate cell concentrations given by the OECD 301 guideline

The OECD guidelines have not specifically described simulation biodegradation test versions that are applicable to volatile chemicals, but depending on the analytical techniques and test setup, volatile chemicals can be evaluated. The OECD 309 guideline specifies that chemicals with Henry’s constants < 1 Pa m^3^ mol^−1^ can be tested in open vessels and chemicals with Henry’s constants < 100 Pa m^3^ mol^−1^ can be tested in closed vessels. These cut-off criteria correspond to equilibrium headspace contents of < 0.1% and < 10% of the test chemical, respectively, if 2/3 of the test system is headspace as suggested in OECD 309. Please note that it is important to avoid using flow-through systems for semi-volatile chemicals, as the test chemical will then be stripped off and lost from the test (Shrestha et al. 2020).

Some simulation biodegradation test modifications for volatile chemicals have been described in the literature. In sediment and soil tests for volatile chemicals, it was found important to reduce the headspace to ensure that the test chemical was present in the soil or water/sediment phase (Shrestha et al. 2019, 2020). The smaller headspace necessitated periodic addition of oxygen to keep the system aerobic, and any air flushed out of the system was passed through a tenax tube to trap test chemicals. Pelagic surface water biodegradation tests (OECD 309 type tests) have also been modified for volatile chemicals, where, to minimize losses, tests were conducted in gas-tight autosampler vials for direct and automated extraction and analysis of substrate depletion (primary degradation) (Birch et al. 2017, 2018).

### UVCBs

Biodegradability is generally seen as an intrinsic property of one substance. This concept is challenged when it comes to UVCBs that are complex mixtures with variable and not fully resolved compositions of numerous constituents (Salvito et al. 2020). According to ECHA Guidance, all known ingredients from UVCBs, at least those present at concentrations ≥ 10%, should be specified during registration (ECHA 2023b). The current guidelines for biodegradability testing generally use non-specific parameters for assessment: O_2_, CO_2_, and DOC (OECD 301), which cannot discriminate between degradation of individual constituents in the UVCB. Furthermore, ^14^C-labelling of UVCBs for simulation biodegradation testing is generally neither possible nor meaningful, but single constituents of the mixture can be evaluated with ^14^C-labelling if available (ECHA 2023b). The main challenge for determining the biodegradability of UVCBs is therefore that standardized tests lack the ability to prove the degradation of all constituents of UVCBs if the constituents are structurally diverse. Another challenge for UVCBs is that they often consist of volatile and hydrophobic constituents. The requirements and modifications previously mentioned may also be relevant for UVCBs.

OECD guidance on ready biodegradability screening tests of mixtures simply states that the theoretical oxygen demand (ThOD) can be calculated from elemental analysis of the mixture (OECD 301 1992), and that for multi-constituent substances the 10-day window as criterion for ready biodegradability is not applied (OECD 2006). This is because the degradation of multi-constituent substances often follows a stepwise degradation sequence with overlapping biodegradation kinetics, which results in “apparent” kinetics that are slower than the individual biodegradation kinetics of the constituents. However, while a standard ready biodegradability test conducted with a UVCB may be able to reveal whether 70% of the mixture (as DOC) is degraded in the test or 60% ThOD is reached, it cannot reveal whether all constituents are degraded at least 70%, and the UVCB could still contain persistent constituents. Therefore, the ECHA guidance on persistence, bioaccumulation, and toxicity (PBT) assessment only allows whole substance biodegradability testing if constituents are shown to be sufficiently similar in structure (ECHA 2023a). If not, tests should be conducted for representative constituents of the UVCB (ECHA 2023a).

The approach taken thus far to enhance whole substance testing of UVCBs involves conducting supplementary analyses to generate more comprehensive data. Brillet et al. (2018) added the measurement of dissolved organic carbon and biomass carbon content to the measurement of carbon dioxide to investigate the carbon mass balance in the test. This does, however, not identify possible persistent constituents. Several recent studies combined whole UVCB tests with constituent specific analysis for determining the biodegradability and persistence of UVCBs (Birch et al. 2024, 2025; Booth et al. 2023). The most recent study demonstrated the simultaneous determination of whole substance mineralization (oxygen depletion) and constituent specific primary degradation kinetics (GC/MS) as two lines of evidence for ready biodegradability of essential oil UVCBs (Birch et al. 2025). The method also addressed the challenges of hydrophobic and volatile constituents. These new possibilities are of course dependent on the availability of suited analytical (target or screening) methods applicable for constituents in the UVCB of interest.

### Conclusions and recommendations for testing hydrophobic chemicals, volatile chemicals, and UVCBs

A methodological gap was identified for ready biodegradability tests with hydrophobic chemicals since ready biodegradability tests are currently conducted above water solubility for many hydrophobic chemicals, which highly complicates interpretation of test results. This methodological gap should be addressed through future scientific developments.

Through a careful consideration of the processes that occur in biodegradation tests with hydrophobic chemicals, the following principles have been identified as important when modifying simulation biodegradation tests for hydrophobic chemicals: (1) to minimize losses by ensuring that no sorptive materials (plastic and rubber) are in contact with the test solution or headspace of the test, (2) to ensure test concentrations are well below solubility, and (3) to account for minor losses by abiotic controls. Such modifications are often needed and justified for hydrophobic chemicals.

The current OECD recommendations for ready biodegradability tests with volatile chemicals are only appropriate if chemicals are not lipophilic. For lipophilic and volatile chemicals, the OECD 301D is the only appropriate test method in the 301 test series. Further methods have been identified that are suitable for the rapid biodegradability testing of volatile chemicals (Brown et al. 2018 and Birch et al. 2025). The general principles for these modifications are (1) to minimize losses during setup by careful handling of aqueous solutions, (2) to minimize losses during incubation by using air-tight and plastic/rubber free test systems, (3) to minimize headspace while ensuring aerobic conditions, and (4) to account for minor losses by using abiotic controls. Simulation biodegradation test modifications have also been developed based on the same principles (Birch et al. 2018, 2023). The authors recommend that these general principles for modifications are deemed justified and adopted in test guidelines.

Biodegradability testing of UVCBs is currently under rapid development. It is also concluded that combining whole substance testing with constituent-specific analysis is an essential step in developing reliable persistence assessments of UVCBs. Furthermore, the combination of whole substance testing with constituent-specific analysis is undoubtedly an important step in developing reliable persistence assessments of UVCBs. While it requires more on the analytical front (development of analytical methods appropriate for the UVCB), the tedious synthesis and testing of constituents one by one is avoided.

## Biodegradation of polymeric compounds

### General aspects about polymers

Polymeric compounds fall into two broad categories: water-soluble polymers (WSPs) and plastics. WSPs are used in detergent and personal care products, water and wastewater treatment, and agricultural products (Kintzi et al. 2024; Royal Society of Chemistry 2021; Vandermeulen et al. 2022). Biodegradable WSPs currently in the market include polyethylene glycol (PEG) (Bernhard et al. 2008; Duis et al. 2021; Kawai 2005; McDonough et al. 2023; Menzies et al. 2023), polyvinyl alcohol (PVA) (McDonough et al. 2023; Menzies et al. 2023; Solaro et al. 2000; van Ginkel and Stroo 1992), and polyaspartic acid (PAsA) (Alford et al. 1994; Freeman et al. 1996; Nakato 1998, Nakato and Kakuchi 2000; Patent EP 0777696B1 1996) to name a few. WSPs are mixtures of structurally diverse high molecular weight (MW) materials, making biodegradation assessments challenging. The breakdown of WSPs is often a two-step process. In the first step, extracellular enzymes break down the polymers to smaller units or monomers, which are subsequently taken up by the microbial cells and then subjected in a second step to the intracellular metabolism with the aim of energy generation of the cells and accompanied by the incorporation into new cellular biomass (Zumstein et al. 2022). Existing technical guidelines (TGs) were not designed to handle these complexities of test substance and biodegradation mechanisms, and new methods are therefore needed.

The annual amount of plastic production in 2023 was more than 400 million tons, and without changes to regulation, plastic production is estimated to reach 800 million tons in 2035 (Barra and Leonard 2018). Major products are polyethylene (PE) and polypropylene (PP), which make up about 50% of the industrial plastic production (Amobonye et al. 2021; Cai et al. 2023). In general, PE, PP, polyvinyl chloride (PVC), and polystyrene (PS) are regarded to be not biodegradable because of their very stable chemical bonds. Biodegradable alternatives to these materials exist, polymers such as polyhydroxyalkanoates (PHAs), polybutyleneadipate-co-terephthalate (PBAT), and polycaprolactone (PCL). Over the past few decades, a number of standards have been developed for the development and certification of these biodegradable alternatives. Some of these standards have also been included in the recent EU polymer microparticle restriction (European Union 2025). However, challenges do remain in the assessment of biodegradable plastic materials. These are due to their complex physical and chemical structure, analytical limitations, and generally slower biodegradation kinetics.

### Biodegradation of water-soluble polymers

By nature of their synthesis pathways, WSPs are typically mixtures of structurally similar species with MWs above 1000 Da (McDonough et al. 2023). Additionally, many WSPs are engineered with specific structural properties that influence their solubility, conformation, and accessibility to enzymes. It is important to note that commonly used TGs such as the OECD 300 series of screening and simulation studies were developed for low MWs, single constituent molecules, and not high MW polymeric mixtures. As previously stated, the biodegradation of WSP differs from that of low MW molecules due to its typically multi-step nature, involving both extracellular and intracellular enzymatic processes. In light of the intricacies inherent in structural properties and biodegradation processes, and given the recognition that the prevailing standardized TGs are not tailored to WSPs, it is necessary to assess the potential for enhancing existing and devising novel test methods specifically for WSPs. This section will discuss the areas of greatest impact on WSP biodegradation predictions, as identified by research. These include inoculum pre-treatment and adaptation, test duration, test chemical to inoculum ratio, and the importance of environmentally realistic test conditions in laboratory assays (simulation studies). While much of the research on test methods for accurately assessing WSPs is in its infancy and more research is needed, some promising discoveries have already been made which will be discussed in the following sections.

### Inoculum pre-treatment and extracellular enzymes

One of the first steps commonly employed in screening biodegradation assays (OECD 301 1992) is the washing and aeration of the inoculum from the wastewater treatment plant, as per OECD guidelines, for 5–7 days before testing. These pre-treatment steps are commonly done to reduce the background levels of CO_2_ production, O_2_ consumption, or DOC from the inoculum. It has been reported that this step is beneficial for the homogenization of the kinetics of the biodegradation between the different test substance replicates (Vázquez-Rodríguez et al. 2007). However, it has also been demonstrated that this pre-conditioning can impact bacterial communities, potentially leading to the loss of specialist degraders and extracellular enzymes that play a crucial role in WSP biodegradation (Kintzi et al. 2024; Vázquez-Rodríguez et al. 2007). According to Kintzi et al. (2024), pre-conditioning steps such as avoiding aeration and washing of the inoculum, incubating with filter-sterilized wastewater, and lowering the test substance concentration can significantly increase biodegradation rates for WSPs. These steps are essential for maintaining extracellular enzyme levels, which are crucial for the effective degradation of WSPs into lower MW fragments. The study revealed that variations in these pre-conditioning steps had a substantial impact on the biodegradation of polylysine and polyaspartic acid, emphasizing the necessity for customized pre-conditioning in WSP biodegradation screening studies. In contrast, small molecules generally do not require tailored pre-conditioning because their simpler structures (especially lower MW) are more accessible to microbial enzymes and less reliant on extracellular enzymes for breakdown.

### Adaptation of inoculum

There are two key areas where adapting inocula to test substances has proven to be advantageous. Firstly, for new, world-first test substances, it is essential for microbial communities to adapt in order to biodegrade. Secondly, for tests being conducted at non-environmentally relevant high test substance concentrations (due to analytical limitations) where competent degrader communities must enumerate to levels high enough to handle the dose concentration. In these two instances, adapted inoculum can be used to more accurately predict the probability that biodegradation will occur under realistic environmental conditions with prolonged exposure of the microbial communities to the new chemistries.

Adaptation of microbial communities present in the environment may be even more important for WSPs as many new polymers are being developed to drive both biodegradability and sustainability goals. There are no studies (which the authors are aware of) that demonstrate field adaptation of microbial communities to new-to-the-world WSPs. However, there are a few studies on chemicals that indicate adaptation may be important for some novel test chemistries. One of the few demonstrations that quantitatively showed field adaptation of microbial communities to a chemical disposed of down the drain was conducted on a chelator (L-glutamate-*N,N*-diacetate, L-GLDA), which is not a WSP, and is used in automatic dishwasher detergent (Itrich et al. 2015). This research showed that when L-GLDA was not in commercial use, it was not biodegradable in OECD 301B (1992) respirometry screening assays by microbial communities present in US domestic wastewater treatment plants. Once L-GLDA was brought into commercial use and microbial communities were continually exposed to the chemical, field adaptation occurred and L-GLDA was shown to be biodegraded in OECD 301B (1992) laboratory screening assays. Adaptation can also be an important component of laboratory studies when, due to analytical limitations, assays cannot be conducted at actual environmental concentrations (typically µg L^−1^ levels or lower), resulting in an adaptation period needed in the laboratory assay to allow time for growth of the competent degrader community to sufficient levels to be able to biodegrade the unnaturally high-test substance concentration. This was shown to be the case in the L-GLDA research when flow-through activated sludge bioreactors seeded with microbial communities from US domestic WWTPs prior to commercial introduction of the chemical were able to biodegrade the chemical after a sustained laboratory adaptation period allowing for proliferation of competent degraders (Itrich et al. 2015). In scientific literature, there are also examples where the pre-adapted inoculum showed lower biodegradation potential (Çelik et al. 2023; Dalmijn et al. 2021; Poursat et al. 2019). Poursat et al. (2019) found within the ECO29 (2019) project that the capability of fresh activated sludge to degrade metformin and guanyl urea, which are not WSPs, was higher than activated sludge pre-exposed to these test compounds in a chemostat system. It is not clear why this occurred, but it should be noted that the chemostat system was not operated under environmentally relevant conditions and the microbial communities shifted significantly during the chemostat exposure.

Analytical method sensitivity is particularly an issue for the quantification of WSP biodegradation, as specific analytical method limits of detection for polymers are typically orders of magnitude higher than actual environmental concentrations, resulting in time needed for the competent degrader community to enumerate sufficiently such that biodegradation can be quantified in a laboratory assay. This issue was demonstrated for polyvinylalcohol (PVA) (degree of hydrolysis (DH) = 88%, MW = 130 kDa) when an OECD 303A-activated sludge simulation study was conducted following DOC removal as the analytical endpoint, which required dosing PVA more than 100 times higher than the actual concentration entering a domestic WWTP. A laboratory adaptation period was needed in the study to allow time for the competent degrader community to proliferate to a level capable of degrading the high-dose concentration of test substance needed for analytical quantification, but once this occurred, high levels of biodegradation were sustained over the remainder of the study (McDonough et al. 2024).

### Extended test duration

As previously mentioned, the complexity of WSPs can result in a slower biodegradation process which may not conform to time frames commonly allotted in standard screening biodegradation studies. Many screening studies have shown that polymers have not reached the full extent of possible mineralization (as evidenced by a plateau in biodegradation) in 28 days, but instead, test duration must be extended to more accurately evaluate the extent of mineralization possible for that polymer. This is an issue because one of the primary purposes of a screening biodegradation study is to evaluate the potential extent of mineralization expected for a test substance in the environment. If the test setup does not allow for this evaluation for some polymers, then new test methods need to be developed. The slow biodegradation of WSPs in screening studies was demonstrated in OECD 301B (1992) studies by Menzies et al. (2023), which examined the biodegradation of high MW WSPs, specifically polyethylene oxides (PEOs) and carboxymethyl celluloses (CMCs). For CMCs, the extent of biodegradation was correlated with degree of substitution (DS); CMC DS = 0.6 exhibited 70% biodegradation but required an extension of the testing period to 148 days for the test substance to begin to reach a plateau in mineralization. If the study had been stopped at 28 days (as per the TG), then the predicted mineralization would have only been 20%. Similarly, CMC with DS = 0.79 reached only 3% mineralization in 28 days but once test extension was employed reached 45% and was still biodegrading at study completion (148 days). Multiple PEOs were also evaluated (MWs of 50, 100, 300, and 500 kDa) and reached < 20% mineralization at 28 days but > 80% mineralization at study completion (148 days). Other researchers have also reported a need for test extension when evaluating WSP biodegradation in screening studies including when evaluating CMCs (McDonough et al. 2023; van Ginkel and Gayton 1996), cationic modified guar gum (McDonough et al. 2023), PEGs (Bernhard et al. 2008), and polypropylene glycols (West et al. 2007) to name a few.

### Moving towards more environmentally relevant test chemical to inoculum ratios

There has been extensive discussion of the need for laboratory methods that move towards more environmentally relevant conditions in terms of test substance concentration and microbial inoculum level, particularly in the context of low MW molecules. Many researchers have highlighted the known issues with standardized screening studies (OECD 301 & 310) which result in false negatives including limited microbial diversity and abundance, inoculum pretreatment techniques, and nonrealistic test chemical to inoculum ratios (Kowalczyk et al. 2015) to name a few. These issues may be even more impactful when assessing WSP biodegradation due to the complexities of the biodegradation process. It has been reported in different WSP biodegradation studies that improving the test chemical to biomass ratio can result in shorter time needed to reach the plateau phase of biodegradation. For varying MW PVAs (DH = 79 and 88%), PEGs and PEOs, and CMC (DS = 0.6) tested in modified screening studies with improved test chemical to inoculum ratios and overall higher inoculum concentration (an increase in microbial abundance and diversity), the time required to reach complete mineralization was shortened for all WSPs, but the improvement was more dramatic for polymers that had been slower to biodegrade in the standard screening assay OECD 301B (1992) including PEO 50 and 100 kDa and CMC DS = 0.6 (Menzies et al. 2023). Building on these observations, Wilcox et al. (2025) evaluated a CMC (DS = 0.65) in a standard OECD 301B (1992) study and a modified study with more environmentally relevant (lower) test chemical mass to inoculum ratios (1:0.5 vs 1:5.6). In the modified study, > 60% mineralization was quantified in 15 days, while in the standardized study, 60% mineralization was not reached by the end of the 70-day study. Wilcox et al. (2025) further showed this same polymer was even more rapidly biodegraded in three different OECD 314B Activated Sludge simulation studies reaching > 60% mineralization in less than 6 h, showing that for this polymer, the standard OECD 301 screening study was too conservative and not predictive of what would happen under more realistic environmental conditions.

### Simulation studies

In the context of simulation studies, the OECD permits the use of more realistic environmental conditions, particularly those related to test substance concentration, inoculum level, and treatment. This approach is intended to facilitate the evaluation of biodegradation rate and extent of biodegradation. These types of assays allow for more precise quantification of parent polymer biodegradation, metabolite formation and decay, and mineralization over the course of a study. They also allow for the corresponding quantification of primary and ultimate biodegradation rates, which are necessary for quantitative environmental risk assessments. These types of assays are of particular importance for WSPs, as they allow for increased microbial diversity, the presence of extracellular enzymes, and more environmentally relevant test chemical to inoculum ratios, as they are conducted under more realistic environmental conditions. There is a lack of WSP simulation study data, but the information collected to date highlights the importance of these types of assays when evaluating polymer biodegradation. One of the earliest studies which highlighted the importance of improving environmental realism in polymer biodegradation assays was conducted by Blanchard et al. (1976). In this research, ^14^C methyl cellulose (DS = 1.9 and degree of polymerization of 430) that had reportedly shown no biodegradation in a 20-day biochemical oxygen demand screening assay, biodegraded extensively in a modified study with higher levels of inoculum. In the modified study, activated sludge inoculum was utilized as received (no inoculum washing) and diluted into dechlorinated water with a resulting inoculum concentration of 5.9 g L^−1^ suspended solids and ^14^C methyl cellulose concentration of 16 mg L^−1^ (significantly higher than would be expected under actual environmental conditions). Despite the higher test substance concentration, significant parent polymer biodegradation (> 75%) and correspondingly ^14^CO_2_ generation (> 60% mineralization) were observed within 10 days with a reported 73% mineralization by 20 days.

As outlined before, Wilcox et al. (2025) conducted a comprehensive evaluation of a ^3^H-CMC DS = 0.65, encompassing both an OECD 301B (1992) type screening study and a multiple OECD 314B (2008) activated sludge simulation study. In the screening study ^3^H-CMC DS = 0.65 was relatively slow to biodegrade, reaching 60% in 40 days, while in all three activated sludge simulation studies, 60% mineralization was reached in less than 6 h. For one of the OECD 314B studies, gel permeation chromatography (GPC) coupled with solid scintillation counting was leveraged to quantify parent polymer and biodegraded polymeric fragments over the course of the study and showed that parent polymer started to biodegrade immediately in the study, with 37% lower MW fragments measured after only 5 min. These studies highlight the need for improving biodegradation screening assays, as multiple examples found that WSPs that showed limited biodegradation in OECD 301 screening studies biodegraded significantly in simulation studies.

In a unique sludge-treated soil simulation study, the degradation and leaching of ^14^C-cationic polyacrylamide copolymer (^14^C-PAM) were evaluated under realistic environmental exposure conditions and polymer concentrations (Hennecke et al. 2018). ^14^C-PAM was shown to slowly biodegrade over the course of the 3-year outdoor lysimeter study. GPC showed that the^14^C-PAM was degraded to lower MW fragments and significantly mineralized (based on loss of total radioactivity) over the course of the study. In another study, the biodegradation of a PEG with a MW of 6000 g mol^−1^ and its photochemically generated degradation products was investigated utilizing ^13^C-labelling, combined with Monte Carlo’s simulations. The process of labelling all carbon atoms of the polymeric chain with isotopes allowed for continuous tracking of mineralization of PEGs and its degradation products to ^13^CO₂ in soil and lake water sediment over several months. This process also enabled closure of the mass balance. Induced chain scissions in PEGs reduced their molecular weight, enhancing biodegradation (Kleemann et al. 2025). While the concentrations investigated in the study are above those typically used for simulations studies and more in line with ISO methods, such as ISO 17556 (2019), ^13^C labelling has the potential to be used for simulation studies, but also for the development of methodologies closing the gap between simple screening and elevated simulation tests.

These investigations exemplify the importance of simulation studies to provide fundamental knowledge on the fate (degradation, biodegradation, irreversible sorption) of WSPs in environmental compartments of concern. While these methods are of the highest relevance, challenges are still linked to the availability of isotopically labelled monomers and polymers the high synthesis costs, especially of the radioactive materials, which force production only on a small scale. While the process of synthesis down-scaling for isotopically labelled analogues of pure chemicals does not substantially affect their properties, it has the potential to impact the molecular weights and distribution, as well as other specific characteristics of polymeric materials. This necessitates a careful characterization of the materials and the development of strategies to investigate exemplary materials and structures and elucidate the transformation of polymers.

### Biodegradation of plastics

The largest share in terms of production volumes for polymeric materials is represented by water-insoluble, structural polymers, normally blended with other polymers and additives to create plastics. Structural polymers, like WSPs, are not directly assimilated by microorganisms for metabolization due to their large size and molecular weight. To be biodegraded, structural polymers and plastics require different stages which include colonization by microorganisms of the surface of the solid material, cleavage of the polymeric chain by extracellular enzymes, uptake by microorganisms, and mineralization of the oligomers and monomers. The most widespread structural polymers are durable materials containing C-C bonds in the polymeric backbone regarded to be not accessible to biodegradation. These include polyethylene (PE), polypropylene (PP), polystyrene (PS), and polyvinylchloride (PVC) (Wierckx et al. 2018). Other structural polymers such as polyesters, polyamides, polyethers, and polyurethanes containing functional groups in the polymeric chain can undergo enzymatic cleavage and successive metabolization. While the chemical structure of the material plays a crucial role, the necessary enzymatic degradation can only occur if the cleavable bonds are also accessible to extracellular enzymes. Therefore, other factors besides chemical structure are decisive such as crystallinity or glass transition temperature. These properties can enhance the durability of materials such as polyethylene terephthalate (PET) or nylon. Alternatively, they can be utilized in the production of biodegradable or compostable products, including polybutylene adipate-co-terephthalate (PBAT), polycaprolactone (PCL), and polyhydroxyalkanoates (Mangold and von Vacano 2022; Torena et al. 2020; Wierckx et al. 2018). Today, knowledge gaps concerning the biodegradation of polymers, including micro- and nanoplastics, remain. Albright and Chai (2021) address in their review certain points which have to be solved in the near future. These concern the establishment of reliable standardized guidelines for testing in different environmental matrices for all types of polymers, an integrated analytical approach using various simple and effective methods and accelerated reliable biodegradation test methods. They also claim the use of predictive methods for the biodegradation of polymers and improved frameworks for the assessment of persistency of polymers. These points have also been emphasized in other publications reviewing the applicability of existing biodegradation methods to synthetic polymers (ECETOC TR No. 133-2 2020; Hahn and Hennecke 2023).

### General considerations for assessing the biodegradation of plastics with standard laboratory methods

Plastics’ biodegradation standards, independently from the inoculum utilized for the test, have several commonalities. First of all, these methodologies investigate the potential of the tested sample to be metabolized by microorganisms from a specific environment into carbon dioxide, biomass, water, and inorganic salts (under aerobic conditions). Before performing biodegradation tests, it is necessary to perform thorough characterization of the material to identify products and analyze the proportion of organic substances, additives, and contaminants present in the test item. Without proper material characterization, which includes the quantification of total organic carbon available for biological degradation, biodegradation results cannot be correctly interpreted.

Currently not covered by existing biodegradation standards is the characterization of other material aspects influencing biodegradation kinetics. Additional characterization would strongly strengthen comparability of results across different studies and more systematically advance the knowledge about biodegradable polymers. An example is molecular weight distributions, which play a key role. The length of polymer chains dictates whether the substrate necessitates extracellular enzymatic cleavage on multiple occasions before it can be utilized by microorganisms for metabolic processes, or whether it can be readily absorbed by microorganisms for metabolic processes.

As plastic items are typically macroscopic objects, samples need to be cryo-milled to micrometer size particles (ideally 100–300 µm) prior to biodegradation testing. While this process appears to reduce the environmental relevance of the methods, this step is necessary to minimize the dependence of biodegradation on unfavorable surface-to-volume ratio limiting substrate availability to microbial degraders. The use of micronized powder also ensures a homogeneous distribution of the sample in the inoculum. This helps to minimize the effects that can result from localized nutrients or microbial degraders.

Biodegradation is typically determined in laboratory tests by analyzing the carbon dioxide released or the oxygen consumed (under aerobic conditions) through microbial degradation of the test material relative to a reference material. As with biodegradation, a fraction of the polymeric carbon is included in the microbial biomass (Zumstein et al. 2018). Pass criteria typically require 90% biodegradation, absolute or relative to a reference material. The use of relative biodegradation in comparison to a reference material is an effective method of accounting for the varying degrees of conversion of polymeric carbon into microbial biomass.

Generally, standard biodegradation tests do not simulate real-world conditions, but they estimate the potential of the tested material to be biodegraded by microorganisms present in the receiving environment. As the test materials’ concentrations are typically above environmentally relevant concentrations, and the inoculum is usually supplied with nutrients to avoid false negative results due to nutrient limitations, these methodologies can be considered screening methodologies. Correlation between laboratory results and more realistic field conditions can be investigated in mesocosm tests and field tests. These methodologies provide a means of determining the biodegradation of materials in the environment. While they have been proven to biodegrade under laboratory conditions, quantitative measurement of the conversion of polymeric carbon into carbon dioxide and biomass is typically not possible.

### Composting tests

Composting processes are based on the aerobic metabolism of various microorganisms (such as bacteria and fungi) that can utilize organic material as a source of carbon and energy, converting it into carbon dioxide, water, and mature compost (Wang and Zeng 2018). Composting tests are aerobic biodegradation tests developed to investigate solid materials, e.g., structural polymers or plastics used in the production of compostable items like bags or packaging for bio-waste collection. In the 1990 s, industry and research institutes developed composting test methods and criteria for testing and labelling of products in accordance with the European Union’s Packaging Regulation (Directive 94/62/EC 1994). The European standards DIN EN 13432 (2000) and DIN EN 17427 (2022) outline a testing strategy for all packaging materials intended for composting. Such testing strategies include material characterization, biodegradation, disintegration, and ecotoxicity testing.

Biodegradation tests for industrially compostable materials are performed at about 58 °C to resemble the conditions found in industrial composting plants (temperatures above 55 °C are reached during the thermophilic active phase). In order to define a material biodegradable under composting conditions, 90% absolute or relative biodegradation versus the reference material must be reached within 6 months, but the test can be stopped as soon as the plateau is reached. The maximal test duration might be considered long compared to the average residence time of compost in technical composting plants (about 40 days). However, as previously mentioned, these tests are designed to assess the biodegradability of the material under the end-of-life conditions. To achieve a high signal-to-noise ratio, the concentrations of testing material used in the inoculum are higher than those actually present in composting plants (Pagga et al. 1995). Furthermore, mature compost from composting plants or aqueous compost extract absorbed by vermiculite is typically used as inoculum (Bellia et al. 1999, 2000; Pagga et al. 1995; ISO 14855-1 2012; ISO 14855-2, 2018). These inocula are much less active than fresh organic waste, but necessary to reduce basal respirometric activity of the inoculum to measure material biodegradability without isotopically labelled materials.

### Anaerobic digestion tests

Anaerobic digestion processes consist of four stages: hydrolysis, acidogenesis, acetogenesis, and methanogenesis. These processes are often performed under mesophilic (35–40 °C) and/or thermophilic (55–60 °C) conditions, resulting in the production of biogas (composed of methane and carbon dioxide) and biomass (Cucina 2023).

Anaerobic digestion of organic waste can be measured using the established ISO 15985 (2014) method, which covers the broad field of anaerobic biodegradation processes. This test method is an optimized simulation of an intensive anaerobic digestion process and determines the complete biodegradability of a sample under high-solid anaerobic digestion conditions. The methanogenic inoculum is obtained from anaerobic digestion tanks containing pre-treated household waste, ideally only the organic fraction. The sample material is mixed with the inoculum and placed in a static digestion vessel. During the anaerobic biodegradation of the sample material, degradation products such as biogas, water, mineral salts, and biomass are produced. The biogas formed is continuously monitored in order to determine cumulative biogas production. The percentage degree of biodegradation is given by the ratio of the amount of biogas produced from the sample material to the maximum amount that can theoretically be produced. This maximum theoretical amount is calculated based on the measured total organic carbon (TOC). However, this percentage value does not take into account the amount of carbon converted into new cell biomass, since this biomass is not metabolized into biogas during the test. It has to be mentioned that this test method is basically the same as the ISO procedure for the determination of anaerobic degradation of organic compounds in aqueous systems (ISO 11734, 1995). Additional standards are presently under development at ISO to broaden the scope of test methodologies for anaerobic biodegradation, ensuring a more accurate reflection of the diverse conditions encountered in full-scale anaerobic digestion facilities.

## Standard methods for biodegradation of plastics in soil and aqueous environments

The conventional degradation methods in an aqueous environment are not designed for testing plastics. All methods based on determining the decrease of DOC are ruled out for analytical reasons only, since plastics are also mechanically eliminated during the necessary filtration or centrifugation of the test samples. It is possible to employ methods that are based on measuring the biochemical oxygen demand (BOD) or the carbon dioxide produced in aqueous systems. However, the biodegradation potential of the inoculum originating from freshwater or WWTPs may be far too low for plastics. Therefore, these methods may not be relevant for plastics whose end of life is in technical composting and digestion plants. In addition, important groups of microorganisms, such as fungi or actinomycetes, which primarily cause the degradation of plastics in soil and compost, are missing in a freshwater environment. For these reasons, neither the OECD biodegradation test methods nor the corresponding ISO standards are suitable for plastics testing, and the criteria of “readily biodegradable” and “inherently biodegradable” are irrelevant. A general comprehensive overview of the available degradation test methods, test criteria and evaluations has been listed in the ISO/TR 15462 (2006) standard and has recently been actualized and summarized (Strotmann et al. 2023).

Tests in an aqueous environment are easier to carry out than in soil or compost and a positive test result can give hints for biodegradability in other environmental compartments. Some existing test methods have already been developed for plastics. The ISO 14851 (2019) respirometric test is based on the well-known and widely used OECD 301F (1992)/ISO 9408 (1999) method, and the ISO 14852 (2021) CO_2_ evolution test is an adjustment of the OECD 301B (1992)/ISO 9439 (1999) method. Not only do these tests require significantly less testing effort than a composting test, but they also have the advantage that the test results can be used to set up a carbon balance and a heterotrophic yield coefficient, which can be important for the interpretation of the test results. Therefore, at the end of the test, the conversion of the organic carbon of the test substance into carbon dioxide, new biomass, and degradation products is quantitatively determined. Testing times of these methodologies have been limited to a maximum of 6 months for technical reasons, but prolongation should be possible to allow more accurate determination of the biodegradability potential.

Degradation tests in soil such as ISO 17556 (2019) play a decisive role for the determination of plastic and polymer degradation, especially for products that enter into soils after application, such as soil biodegradable mulch films or fertilizer coatings. Such methodologies are already anchored in product specific standards, such as the ISO 23517 (2021) or the EU fertilizer regulation (EU 2024/2770 2024). In recent years, as a response to the pressing discussion on biodegradability of plastics in the marine environment, several standards have been developed to test marine biodegradability of plastics under laboratory conditions in water (ISO 23977-1 (2020) and ISO 23977-2 (2020)), water sediment (ISO 18830 (2016) and ISO 19679 (2020)), and sediment systems (ISO 22404 (2019) and ISO 22766 (2020)). Field tests (ISO 15314 (2018) and ISO 22766 (2020)) and tank tests (ISO 23832 2021) underwent standardization to complement laboratory tests and provide reliable methodologies to correlate laboratory to more realistic field conditions.

For all these methodologies, several challenges remain. The first derives from the long testing times, typically up to 24 months or even longer, which derive from the need to allow sufficient time to assess the biodegradability of the material under the stringent pass criteria. To reduce testing times in soil biodegradation tests, Será et al. (2020) have proposed a methodology suggesting to increase testing temperature from 25 to 37 °C, especially to assess biodegradability of slowly degrading materials. Such methodology has been already included in the revision of the EU fertilizer regulation, but no other studies have been reported utilizing this methodology. The second challenge linked to all tests utilizing environmental samples, but especially for marine biodegradation tests, is the strong variability in the activity of the inoculum. Research to minimize such effects suggests the use of wet filtration and seawater flotation to improve the reproducibility of test results. More robust biodegradation results for cellulose and polycaprolactone were generated by Taguchi et al. (2025) applying this methodology and resulted in the standard ISO 16623 (2024). Finally, methodologies developed for the testing of biodegradable plastics utilizing freshwater inoculum such as sediment or water sediment are still missing.

### Advancements in plastic biodegradation research

In recent years, efforts have been taken to complement existing biodegradation standards to better understand the biodegradation of structural polymers and plastics in different environmental compartments. In particular, methodologies have been developed with the aim of addressing the existing gaps in the field of biodegradation methods. These gaps pertain to the necessity of the following: (1) conducting tests under more environmentally relevant concentrations; (2) selectively tracking polymeric carbon in order to elucidate the inclusion of polymeric carbon into biomass; and (3) identifying and quantifying microparticle formation during biodegradation. Stable carbon isotope labelling has been proven to be a useful methodology to move to more environmentally realistic concentrations and to understand the biodegradation of different monomers or oligomers (Nelson et al. 2022; Zumstein et al. 2018). Combining different analytical techniques, it is possible to close the mass balance and to differentiate between carbon included into biomass and residual polymer. A more detailed analysis about the benefits and limitations of isotopic labelling for polymers has been recently published (Adeleh et al. 2024). Solvent extraction protocols have been applied to soil matrices to quantify the biodegradation of plastics without the need to track mineralization of the material, providing information about the changes in monomer composition and changes in MW distribution (Cerri et al. 2025). In parallel, methods for the quantitative extraction of plastic microparticles transiently formed during biodegradation have been developed and applied to different materials during standardized biodegradation tests. By tuning oxidation and density separation protocols, it is possible to determine the amount, shape, and nature of the fragments generated during biodegradation (Pfohl et al. 2022; Wohlleben et al. 2023). These methodologies are examples of techniques which could allow investigation of biodegradability under more environmentally realistic scenarios and at the same time provide more detailed information about the transformation processes occurring during biodegradation by analyzing polymer-specific endpoints.

### Microplastics and polymeric particles

Microplastics and polymer microparticles are an additional class of polymeric materials that have been the subject of extensive research in recent years (Thompson et al. 2024). Microplastics can be subdivided into primary and secondary microplastics.

Primary microplastics are materials introduced to the market in various products in the form of microparticles measuring less than 5 mm. The EU restriction on polymeric microparticles (EU 2023/2055 2023) has established a scheme for proving the degradability of primary microplastics and other particles containing polymers (see Appendix 15 of the EU restriction). Materials should be tested in the form in which they are brought to market, or in a form that is comparable in size and surface area to that of the original product. The permitted test methods are organized into five groups. Group 1 consists of screening tests for “ready biodegradability” (OECD 301B, 1992; OECD 301C 1992; OECD 301D 1992; OECD 301F 1992; OECD 310 2014). Group 2 consists of modified and enhanced screening tests (eRBTs), as well as OECD 306 (1992) tests, which have an extended test duration of up to 60 days and larger test vessels. Group 3 refers to inherent screening tests. Group 4 comprises screening test methods for determining the biodegradability of the aforementioned plastic materials (ISO 14851:^2019^; ISO 14852:^2021^; ISO 17556:^2019^; ISO 18830:^2016^; ISO 19679:^2020^; ISO 22404:^2019^). Pass criteria for Group 4 tests refer to ultimate degradation of ≥ 90%, relative to the degradation of the reference material, within 6 months in aquatic tests or 24 months in soil, sediment, or water/sediment interface tests. Group 5 comprises several simulation test methods. When using group 4 or 5 test methods, synthetic polymer microparticles must demonstrate biodegradability in three environmental compartments (water, water-sediment, and soil) independently of the products’ actual environmental fate.

Despite the existence of a testing scheme, data assessing the applicability of biodegradation test methods to primary microplastics and polymeric microparticles remains scarce. McDonough et al. (2017) used an OECD 301B test system to determine the biodegradability of poly3-hydroxybutyrate-co-3-hydroxyvalerate (PHBV). They observed that the microparticles could be degraded by 66% within 28 days. Microparticles from jojoba wax, beeswax, rice bran wax, stearyl stearate, blueberry seeds, and walnut shells were also successfully tested. This indicates that the system is well suited to this type of biodegradation test. However, certain handling procedures and an extension of the test duration to 80 days are necessary for reliable performance. In a recent study, Teggers et al. (2025) tested the biodegradation of a ^14^C-labelled polyurea (PUA) capsule suspension (CS) with different particle sizes and determined the ^14^CO₂ liberation. PUA is known to be rigid and stable. As an inoculum, a filtered activated sludge suspension was used. A bulk sample of the material revealed 25% biodegradation (^14^CO₂ liberation) after 161 days of incubation. The authors also emphasize that the test material must be purified and that its size distribution must be measured prior to testing. They also advocate the use of ^14^C-labelled polymers to distinguish polymer degradation from the degradation of other constituents. Given the strict criteria and the fact that the proposed methodologies have not yet been validated for the category of products regulated by the restriction, a significant hurdle has been set for material development. Further research is needed to identify limitations of the current tests and required adaptations.

Secondary microplastics are formed as the result of the degradation of larger plastic objects. Secondary micro- and nanoplastics (MNP) from conventional non-biodegradable polymers are typically generated in the environment as a result of physicochemical processes such as grinding, abrasion, UV-light modifications, and aging. As outlined in several recent publications (Aralappanavar et al. 2024; Hao et al. 2023; Huang et al. 2019; Yu and Flury 2021), the transport of these particles and their impact on microorganisms is a subject that has been the focus of much recent research. As detailed in the report by Gambardella et al. (2019), a thorough investigation into microbial toxicity has been conducted. With regard to these microbial toxicity tests, the focus was often on marine bacteria such as *Aliivibrio fischeri*. The test with this bacterium is a widely used, standardized test system (Araújo et al. 2005; ISO 11348, 2007). However, challenges are being faced in investigating the fate and impact of secondary microplastics from conventional non-biodegradable polymers. These challenges are linked to the variability of the materials that have been tested, the characterization of the materials (including additives), and the limited availability of reference materials.

### Conclusions and recommendations about biodegradation of polymers

TGs such as the OECD 300 series of screening and simulation studies are commonly used for chemicals with low MWs, single constituent molecules, and not high MW polymeric mixtures. When testing the biodegradability of water-soluble polymers, plastics, or microparticles, it is compulsory to consider that such polymeric materials are composed of mixtures of molecules with varying molecular weights. Additionally, high molecular weight polymeric molecules generally require prior cleavage by extracellular enzymes for direct uptake by microorganisms to occur. The biodegradation of polymers is determined by their molecular weight (MW) and MW distributions. This process typically occurs more slowly and is controlled by an extracellular enzymatic cleavage step.

The maturity of research into biodegradability and the availability of relevant data varies between water-soluble polymers, plastics, and microplastics. As outlined in this review, the testing of biodegradation for water-soluble polymers is still in its early stages and will require the generation of data in parallel to the adaptation of existing methodologies or the development of new test methods. Research on biodegradable plastics has evolved over the past decades, and ISO biodegradation standards provide a solid framework for assessing their biodegradability. These screening methodologies still require extended testing periods, and variations in test results depending on inoculum pretreatment and sampling remain a challenge. For polymer microparticles, methodologies still need to be validated in parallel to the development of biodegradable materials that fulfil the requirements of the recently published restriction.

It is recommended that future research initiatives focus on the following key areas. The development of appropriate inoculum sampling and pretreatment methods is essential for accurate assessment of biodegradability. Moreover, the extension of testing periods is instrumental in ensuring the reliability of results. Conducting tests at various test substances to inoculum ratios is crucial for a comprehensive evaluation.

It is vital to acknowledge the pivotal function of extracellular enzymes and degrading microorganisms in the context of polymer biodegradation. Future research endeavors in this field must prioritize a more profound exploration of the underlying microbiological processes, thereby complementing the existing standardized methodologies. In conclusion, it is necessary that further development of methodologies is undertaken for all materials, with the objective of testing at more environmentally relevant concentrations and/or under more realistic conditions. In order to facilitate such development, there is a requirement for analytical methodologies capable of identifying residual polymers and transformation products in environmental matrices. Methodologies that leverage isotopic labelling, whether introduced via custom synthesis, post-modification (Wilcox et al. 2025), or the use of stable isotope labelling (Zumstein et al. 2018), will play a key role. However, these methodologies face challenges in matching the properties of large-scale industrially produced polymers. Consequently, it is not feasible for biodegradability assessment under environmentally realistic conditions to be conducted for all polymeric materials. The development of a testing framework leveraging data from exemplary samples is therefore most likely to be required.

## Characterization of inocula for biodegradation tests and the prediction of biodegradability

### Characterization of inocula

In all biodegradability tests, the inoculum is extremely important but, on the other hand, difficult to characterize and standardize. Therefore, it is often variable and has been designated by some researchers as a kind of “black box” (Kowalczyk et al. 2015; Thouand et al. 2011). Up to now, there exist only a few methods to describe and ensure the quality of the inoculum. One possibility is a clear indication of the inoculum source, amount, and any pre-treatment such as washing, starving, and preadaptation to the test substance. Also, the parallel use of different reference compounds may characterize the physiological properties of the bacterial community and be used to guide interpretation of biodegradation test results. In recent reviews, physiological parameters were promoted to establish a better characterization of the inoculum (Strotmann et al. 2023, 2024). The OECD 301 test guidelines (see Table 2) set out the maximum inoculum concentration permitted in the various tests (in suspended solids per volume) and the corresponding approximate cell density (in colony-forming units (CFU) per volume). This is indicated as being within the range of 10^4^ to 10^8^ cells per liter, depending on the test used. However, there is neither an indication on how the cell number has been determined nor a reference to any standard. Furthermore, Gartiser et al. (2022) found that the CFU determination of the inoculum according to ISO 6222 (1999) (nutrient agar plate method) mainly applied for drinking water, cooling water, or surface water does not provide an accurate estimate of the inoculum activity, especially when used for activated sludge flocs. In addition, modern genetic techniques including 16S rRNA analysis can be used to gain insight into the structure of the bacterial community (Zhang et al. 2018). But these techniques have not yet been standardized, although they are potentially useful for biodegradability studies.

There are a variety of sources for inocula, which contain a diverse range of bacteria. These include seawater, freshwater, activated sludge, and bacterial consortia found in anaerobic treatment and composting plants. Inocula are regarded to be one of the most crucial points when it comes to performing a reliable biodegradation test. The quantity and the quality of inocula are both important. The quantity of inocula can be established through various methods, ranging from the estimation of colony-forming units (CFU) to advanced techniques such as epifluorescence microscopy (EPF), fluorescence microscopy (FCM), and flow cytometry. Alternatively, suspended solids (SS), mixed liquid suspended solids (MLSS), and volatile suspended solids (VSS) can be determined, with these parameters often being comparable. For OECD 301 (1992), OECD 310 (2014), and OECD 302 (1981) tests, inocula are often derived from activated sludge taken from municipal wastewater treatment plants. Important parameters of these inocula are summarized in Table 4. It should also be noted that the OECD 310 TG provides approximate estimates of inocula concentrations through the calculation of CFU (OECD 310 2014). This approach results in significantly lower bacterial concentrations compared to methods based on EPF and FCM. As a comparison, it can be noted that the bacteria concentration as determined by CFU only makes up 0.4 to about 4% of the bacteria concentration determined by EPF (Thouand et al. 1995). In the OECD 310 test system, the bacteria concentration in activated sludge is in the range of 2.5 × 10^7^ to 2.5 × 10^10^ cells g^−1^ VSS (OECD 310 TG, determination by CFU). Concerning the OECD 301 (1992) tests, the estimation of bacterial cells is in the range of 3.3 × 10^8^ to 3.3 × 10^9^ cells g^−1^ VSS. It is important to note that these data are relatively low. In contrast, data obtained by modern methods, such as EPF and FCM, give bacterial concentrations in the range of 10^12^ to 10^13^ cells g⁻^1^ VSS. However, it is necessary to consider not only the total concentration of bacteria, but also other factors that may contribute to the overall quality of the sample. It is crucial to recognize that not all bacteria are capable of metabolic activity. Consequently, it can be deduced that the physiological activity, encompassing the capacity for chemical degradation, is a pivotal factor that warrants meticulous consideration in forthcoming developments. In this context, the concept of the biodegradation adaptation potential (BAP) will be very useful (Strotmann et al. 2023). This concept describes the physiological potential of an inoculum. The concept will be discussed further in a subsequent section of this chapter.

Concerning this point, an update of the OECD estimations seems to be necessary. It should be noted that the OECD screening guidelines do not currently consider food-to-biomass (F/M) ratios, which can play an important role. These ratios are important for the operation of wastewater treatment plants and are a hint for an underload or overload of a technical plant (Hamza et al. 2018). In wastewater engineering, F/M ratios are indicated as a load of COD or BOD per biomass and day. As OECD 301/310/302 ready and inherent biodegradability tests are conducted with the test substance being the dominant food source due to inocula pretreatment (sludge washing), low inocula levels in the study, and no additional food added to the test system, it is interesting to consider the F/M ratios of these studies compared to WWTPs. In instances where the pertinent OECD test is of a static nature and involves solely an initial application of a substrate, it is only possible to calculate an initial F/M ratio. As shown in Table 4, the initial F/M ratios for different OECD biodegradation tests have been calculated. It is evident that these ratios span a considerable range, from 0.25 g DOC g^−1^ VSS in a Zahn-Wellens test to 4348 g DOC g^−1^ VSS in an OECD screening test with an extremely low inoculum concentration. By way of comparison, the typical range for a wastewater treatment plant is between 0.133 g DOC g^−1^ VSS d^−1^ and 0.267 g DOC g^−1^ VSS d^−1^ for conventional aerobic activated sludge processes (CAS) and up-flow anaerobic sludge blanket (UASB) reactors, respectively. For extended aeration processes, the range is from 0.037 g DOC g^−1^ VSS d^−1^ to 0.082 g DOC g^−1^ VSS d^−1^ (Hamza et al. 2018; Sperling 2007). Therefore, it is obvious that F/M ratios should be considered in future revisions of the OECD 301 TG to potentially increase harmonization and improve reproducibility of the screening studies. As a proposal, these calculations indicate that the initial F/M ratios for screening biodegradation tests should lie in the range of 0.1 to 0.3 g DOC g^−1^ VSS. This is partially realized in OECD 301A (1992), OECD 301B (1992), and OECD 302B (1992) test systems when testing in the lower concentration range. However, the F/M ratio is also determined by the signal-to-noise ratio of measurements in the test vessels compared to that of the inoculum blank vessels, and in the closed bottle test (OECD 301D, 1992), it is also influenced by the limited solubility of oxygen in water.

**Fig. 3:**
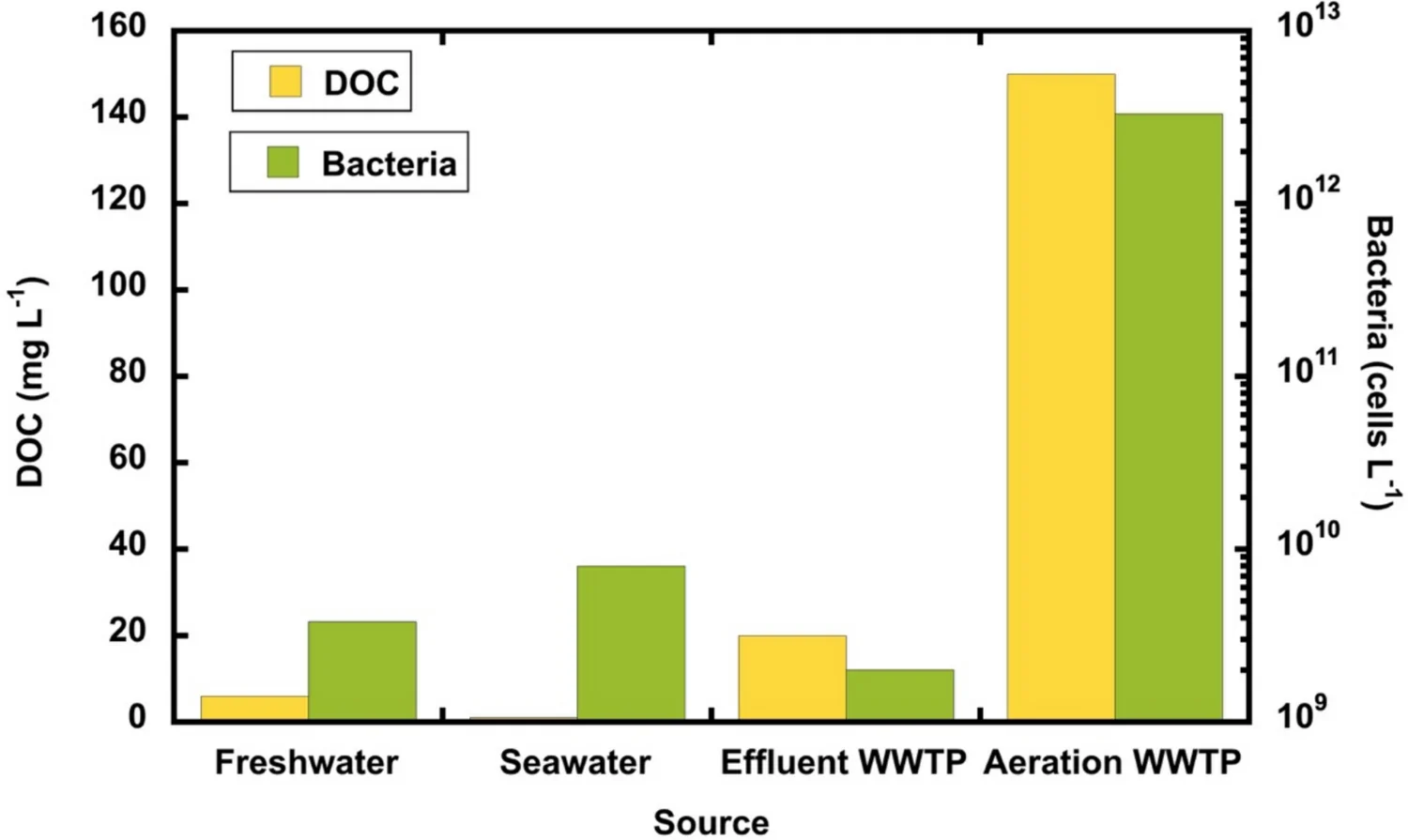
Exemplary DOC concentrations and bacterial concentrations in different sources of water bodies (freshwater, seawater, effluent of a municipal WWTP, and aeration basin of a municipal WWTP). The data demonstrate the magnitude of the typical concentrations of dissolved organic carbon (DOC) and bacteria. All bacterial concentrations are based on EPM measurements. The data were taken from Escalas et al. (2003), Foladori et al. (2010), Liu and Wang (2022), Tchobanoglous et al. (1991), Thouand et al. (1995), and Yoon and Rosson (1990). Further relevant data concerning bacteria in wastewater treatment plants are provided in Table 6

In order to gain a general overview, it is important to understand the range of DOC and bacterial concentrations in various water sources, such as freshwater, seawater, and sewage treatment plants (see Fig. 3 and Table 6). In order to facilitate the process of biodegradation, it is essential that the concentration of competent bacterial cells, which are capable of degrading the specific organic compound under consideration, is sufficiently high. As asserted by Kowalczyk et al. (2015) and Thouand et al. (1995, 1996, 2011), the concentration of competent bacteria must be maintained within the range of 2.5 × 10^4^ to 3.5 × 10^5^ cells L^−1^ to ensure effective biodegradation processes. In addition to the concentration of competent cells, the total number of cells has also been demonstrated to be of significance. An increase in total biomass concentration was found to improve the chances of biodegradation processes (Painter 1995). A brief summary of further technical modifications to improve the test design is provided in Table 5.

**Table 5:** Possible improvements for various biodegradation tests concerning test design and inoculum characterization

	References	Remarks
**A. Test design**		
Multicomponent test system (MCTS)/combined DOC CO_2_ test system/ultimately transformed organic carbon system (UTOC)	Brillet et al. (2018); Gartiser et al. (2023); Norr et al. (2001); Strotmann et al. (1995, 2004)	High reliability of test results, high apparatus demand
Larger test volume in respirometry tests	Nyholm et al. (1992); Painter (1995); Strotmann et al. (2023)	Higher reliability of test results
Preadaptation of the inoculum (RBTs, “Concawe” test)	Battersby et al. (1999); Thouand et al. (1996)	Higher biodegradation potential; does not meet OECD guidelines for RBTs and inherent tests
Extended test duration (enhanced RBT, “Concawe” test) also as needed for WSP assessments	Battersby et al. (1999); Gartiser et al. (2022); McDonough et al. (2023); Menzies et al. (2023); Wilcox et al. (2025)	Exclusion of false negative results; does not meet OECD guidelines for RBTs and inherent tests, but may be used for persistency assessment. WSPs have been shown to need longer test durations
Mix of OECD 301D and 301 F analytical techniques	Brown et al. (2018)	Used for volatile and hydrophobic chemicals
^13^C labelling techniques and ^13^CO_2_ quantification	Kleemann et al. (2025); Nelson et al. (2022)	To fill the gap between ready biodegradation tests & simulation tests
Improved inoculum to test chemical ratios	Menzies et al. (2023); Wilcox et al. (2025)	Critical for WSP evaluations as improved biodegradation profiles observed
Increased cell concentrations	Martin et al. (2017); Ott et al. (2020)	Higher reliability, shortened testing times
More realistic test conditions (simulation type studies)	Blanchard et al. (1976); Hennecke et al. (2018); Wilcox et al. (2025)	Critical for WSP evaluations as improved biodegradation profiles observed
Integrated test strategies	Strotmann et al. (1993a, 2023)	Useful for chemicals failing in ready biodegradability tests, but are potentially biodegradable
Probabilistic modelling	Brillet et al. 2016 Thouand et al. 2011	Useful addition to ready biodegradability tests in order to better estimate biodegradation under realistic environmental conditions
**B. Inoculum characterization**		
Epifluorescence microscopy combined with flow cytometry for the determination of accurate biomass concentrations	Brown et al. (2019); Foladori et al. (2010)	Much more modern technique than the determination of VSS and SS; relatively high costs
Determination of defined physiological parameters of the inoculum (biodegradation adaptation potential (BAP), chemical resistance potential (CRP), physiological potential of an inoculum (PPI))	Strotmann et al. (2023, 2024)	Easily performable; provides a quality control for the inocula and if used helps interpretation of test results
ProbaBio concept	Brillet et al. (2016); Thouand et al. (2011)	Very sophisticated concept; enhances reliability of test results
Community-Level Physiological Profiling (CLPP)	BIOLOG (2025)	Provides important additional information
Use of suitable reference compounds	Comber and Holt (2010); Strotmann et al. (2023)	Can aid in the interpretation of test results
Standardized inocula	Goodhead et al. (2014); Vázquez-Rodríguez et al. (2011)	Can improve reproducibility; concept should be further explored

*https://www.biolog.com/products/community-analysis-microplates/ecoplate/ (17.03.2025)

As previously mentioned, pre-adaptation processes have been shown to significantly improve biodegradability test outcomes (Strotmann et al. 1993a; Thouand et al. 1996; Toräng and Nyholm 2005). In general, inocula from industrial wastewater treatment plants, which treat wastewater from chemical production processes, are often much better adapted to a variety of organic compounds than inocula from municipal wastewater treatment plants (Painter 1995). However, according to the OECD 301 screening study TG, inocula from industrial wastewater treatment plants are not permitted for ready biodegradability tests (Table 6).

**Table 6:** Some important facts about bacteria in wastewater treatment plants

Fact	Parameter	Reference
Biovolume of a single cell	0.16 (µm)^3^ 0.25 (µm)^3^	Foladori et al. (2010); Frølund et al. (1996)
Biovolume of a small cell aggregate in activated sludge (2–3 cells)	0.43 (µm)^3^	Foladori et al. (2010)
Cell conc. in raw wastewater	2 × 10^11^ cells L^−1^	Foladori et al. (2010)
Cell conc. in activated sludge in a WWTP	3.3 × 10^12^ cells L^−1^ 1.08 × 10^11^ – 3.94 × 10^12^ cells L^−1^ (FCM)^1^ 1.69 × 10^11^ – 1.11 × 10^13^ cells L^−1^ (EFM)^2^	Brown et al. (2019); Foladori et al. (2010)
Number of cells in 1 g of VSS	10^12^ – 10^13^ cells g^−1^ VSS^3^	Nielsen et al. (2004)
Cell conc. in the effluent of a WWTP	2 × 10^9^ cells L^−1^	Foladori et al. (2010)
Activated sludge conc. in an aeration basin	2.5–3.5 g VSS L^−1^ 3.05 g VSS L^−1^	Foladori et al. (2010); Tchobanoglous et al. (1991)
Carbon content of cells	53% of dry weight of cells	Foladori et al. (2010)
Relation cells and VSS	1 cell represents about 9.2 × 10^−13^ g VSS^3^; 7.9 × 10^−13^ g SS^4^	Own calculation, Thouand et al. (1995)
Ratio CFU^5^/EPM^2^	1.2–4.1%	Thouand et al. (1995)

^1^*FCM* fluorescence microscopy, ^2^*EPM* epifluorescence microscopy, ^3^*VSS* volatile suspended solids, ^4^ SS: suspended solids, ^5^ CFU: colony forming units

Conversely, adaptation processes may also occur in the natural environment (Toräng and Nyholm 2005). It is evident that both the adaptation processes and the total incubation time are significant factors in the overall process. It is evident that as the incubation time increases, there is a concomitant increase in the likelihood of biological degradation processes occurring. For morpholine, Strotmann et al. (1993a) demonstrated that an adaptation phase of around 16 days was necessary before biological degradation processes commenced. This long lag phase is followed by the actual biological degradation phase. If the total test duration is too short, the test will produce false negative results. For OECD 301-based screening tests, the total test duration is frequently restricted to 28 days, which may be inadequate for certain tests. Consequently, the extension of the test period will facilitate the acquisition of further information regarding potential biological degradation processes. The employment of tools inherent to a pre-adaptation, in conjunction with the utilization of an extended incubation period, is expected to be of considerable utility in the near future in the assessment of the biodegradability of challenging compounds, including hydrophobic and volatile compounds, UVCBs, and polymers. However, it is also true that unrealistically protracted incubation periods do not reflect realistic environmental conditions. Nevertheless, they constitute a convenient method of distinguishing between potentially biodegradable and non-biodegradable organic compounds.

As previously stated, a considerable number of the factors under discussion are inextricably linked to the inoculum used and its origin. An effective way to characterize inocula is to measure their physiological potential. This potential should be determined when using a new or unknown inoculum. This can be achieved by assessing the capacity to degrade a range of common test compounds (Reuschenbach et al. 2003) and by evaluating the biodegradation adaptation potential (BAP). BAP can be used to characterize an inoculum’s ability to adapt to specific, difficult-to-degrade organic compounds (Strotmann et al. 2023). It describes three classes of inoculum depending on their ability to adapt to organic compounds. Thus, quality control of inocula can be introduced to ensure that those used in biodegradation tests meet previously defined standards. The concept can also be extended by requiring the inoculum to have a fixed chemical resistance potential (CRP). This ensures that the inoculum is not inhibited by the concentrations of organic compounds used in biodegradation screening tests (Strotmann et al. 2024). The BAP and CRP concepts can be combined to describe the physiological potential of an inoculum (PPI) (Strotmann et al. 2024). The idea is to guarantee a certain physiological quality of the inoculum used, as this is the most variable factor in many biodegradability tests. In practice, the BAP can be estimated using a reference compound that meets the class 2 or 3 criteria (as defined by Strotmann et al. 2024), such as aniline or diethylene glycol. To estimate the CRP, it is sufficient to determine respiratory inhibition according to ISO 8192 (2007) and/or nitrification inhibition according to ISO 9509 (2006) using 3,5-dichlorophenol in the EC_50_ range (Strotmann et al. 2024, 2023).

Another promising concept for characterizing inocula is the ProbaBio system, which uses a probability-based approach (Brillet et al. 2016; Thouand et al. 2011). This method involves diluting the inoculum to estimate the critical concentration required for biodegradation processes to occur. Furthermore, it can be used to evaluate inocula’s ability to degrade specific reference compounds, thereby estimating their physiological quality. This method should only be used for new, unknown inocula. A closely related concept was developed by Comber and Holt (2010), who created a multi-level list of reference compounds ranging from easily to non-degradable. These reference compounds can be used to determine the physiological potential of inocula in biodegradability tests.

An alternative approach to investigating biological degradation processes involves conducting resting cell experiments with suitable inocula, whereby cell growth is inhibited by restricting the nitrogen or phosphorus source in the incubation medium. This process has been found to inhibit the growth of competent cells, thereby facilitating the investigation into whether biodegradation processes are enzyme induction-based (Strotmann and Röschenthaler 1987; Strotmann et al. 1990, 1993b). While this method is not suitable for screening purposes, it is ideal for more demanding biodegradability tests. Consequently, this technique is becoming increasingly important and could be a promising technology for the future (Ivanova et al. 2022; Nor Suhaila et al. 2019; Seong et al. 2024).

### Prediction of biodegradability

Biological degradation processes in the environment are very complex and are influenced by a number of different factors. Furthermore, chemical concentrations are frequently low, and the substances under consideration are often mixed with other organic material. Therefore, it can be deduced that the degradation processes may also be influenced by co-metabolic processes. It is evident that not all biodegradation testing procedures are designed to accommodate the variability of different environmental conditions (Toräng and Nyholm 2005). This issue also pertains to the evaluation of persistence through biodegradability assessment guidelines (Brillet et al. 2016). It is therefore essential to develop innovative approaches in order to address this issue.

A first approach focuses on QSAR models (quantitative structure-activity relationships, in silico models) to estimate biodegradability. Several QSAR models have been tested with a large dataset, including VEGA, TOPCAT, BIOWIN V.5 and V.6, and START (Pizzo et al. 2013). The models demonstrated a high degree of accuracy in differentiating between “ready biodegradation” and “not ready biodegradation,” with a range of 81–99% (Strotmann et al. 2023). QSAR models can be utilized to differentiate between compounds that are likely to be readily biodegradable and those that are not. Therefore, the first group should be tested using simple RBTs, while the latter group should be tested using enhanced RBTs, inherent biodegradation tests, or even simulation tests (Strotmann et al. 2023). In recent years, QSAR models have attracted increased attention, with significant progress being made in this field to provide more accurate predictions, including in the field of environmental science. Furthermore, realistic models require consistent experimental data produced in improved test systems (Chen et al. 2026a). Chen et al. (2026b) provide a comprehensive overview of this continually expanding field, which will also have a significant impact on the prediction of biodegradability of organic compounds in the environment.

Another possibility would be the establishment of an integrated test strategy. This strategy would consist of a battery of microbial toxicity tests (e.g., luminescent bacteria test, respiration inhibition, and nitrification inhibition), screening tests for ready biodegradability, inherent biodegradability tests, and a subsequent WWTP reactor test also simulating shock loading situations. This strategy has enabled the establishment of a realistic database for an environmental evaluation of morpholine without exaggerated technical effort (Strotmann et al. 1993a). It is submitted that such an integrated strategy may consist of variable appropriate test systems that complement each other. This testing strategy may be employed in the future for a number of organic compounds for environmental predictions.

However, there remains a discrepancy between laboratory tests and reliable environmental predictions. A third approach focuses on closing this gap and includes a probabilistic model to predict biodegradation, including persistence under different environmental conditions (Thouand et al. 2011; Brillet et al. 2016). The ProbaBio model has been tested with a number of organic compounds, including sodium benzoate, 4-nitrophenol, diethylene glycol, 2,4,5-trichlorophenol, atrazine, and glyphosate. The results of these tests demonstrate the model’s effectiveness. The critical inoculum concentration necessary to allow biodegradation is estimated using a dilution method. It is also important to note that the ProbaBio method has not been designed to replace OECD guidelines. Furthermore, it is regarded as offering an additional approach for the assessment of chemical biodegradability using various sources of microbial inocula and temperature regimes (Brillet et al. 2016).

### Conclusions and recommendations for the characterization of inocula and prediction of biodegradability

The characterization of the inoculum constitutes a pivotal aspect in the enhancement of standardized biodegradability tests. In accordance with prevailing OECD and ISO standards, the provenance of the inoculum, along with its concentration, remains the sole piece of information that is often indicated. The practicability of an inoculum is determined exclusively by the use of reference compounds. However, it is important to note that the biodegradation potential of an inoculum may vary significantly, which could have a substantial impact on the results of biodegradation tests. To date, no physiological parameters have been utilized that can provide information on the activity of an inoculum. However, it is imperative to employ such parameters to avoid erroneous test results. It is therefore recommended that (1) the inoculum used for biodegradation tests should be characterized by physiological parameters such as the BAP, CRP, and PPI, and (2) further research should be conducted on new more demanding reference compounds that may be useful for future developments (Strotmann et al. 2023; Schofield et al. 2025). In addition, it is compulsory that these advancements are incorporated into the revisions of the prevailing OECD and ISO standards.

In the context of predicting biodegradability, particular attention must be paid to the following three areas. The enhancement of adequate QSAR models is of paramount importance. To date, the majority of QSAR models are capable of distinguishing between chemicals that are likely to be “readily biodegradable” and those that are not (Pizzo et al. 2013). It is necessary that these models be further refined in order to facilitate predictions regarding the length of lag periods and to enable estimation of the final extent of biodegradation. Furthermore, there is an urgent need to develop integrated test strategies and enhanced, suitable test systems in order to improve the quality of results obtained in biodegradation tests. The results of these improved tests are essential for the creation of future QSAR models. Finally, the utilization of probabilistic approaches, such as the ProbaBio model, is essential (Brillet et al. 2016). It is submitted that these approaches, when considered as a whole, will facilitate a more realistic estimation of biodegradation in the environment.

## New challenge and future approach: proposals for novel methods and approaches to expand biodegradation assessments for difficult to test substance

In order to facilitate progress in the field of science, it is necessary that novel methods and approaches are developed which serve to expand the knowledge of the biodegradation of chemicals, with a particular focus on substances which present significant challenges in terms of testing. It is also necessary to transcend the confines of conventional standard test methods in order to formulate novel approaches which facilitate expeditious, precise, and comprehensive assessment of biodegradation. The development of a comprehensive suite of tools is essential for implementing diverse testing strategies. This review has highlighted a number of areas where changes in methods and approaches have led to significant improvements in the assessment of biodegradation.

The following sections provide concise overviews of the most pertinent demand in key areas, including test substances, analytical aspects of biodegradation, inoculum characterization, enhancement of laboratory assays, and the weight of evidence assessment of biodegradation.

### Test substance synthesis

While not often considered in biodegradation assessments, the ability to synthesize representative, well-characterized test substances for biodegradation assessments is critical, especially for polymeric substances. This includes the ability to synthesize labelled materials (stable or radiolabelled), which allow for improved quantification in biodegradation assays. Little research is currently focused on this scientific area. Furthermore, the ability to evaluate biodegradation in environmental simulation studies is changing our understanding of the biodegradation potential for certain test substances, as well as providing fundamental primary and ultimate biodegradation rate information. As a consequence, there is a need for test substances which specifically enable an improved understanding of biodegradation, especially for difficult-to-test substances.

### Analytical quantification of biodegradation

A significant proportion of extant methodologies for the assessment of biodegradation are predicated on a single, non-specific analytical endpoint (e.g., O_2_ consumption, CO_2_ evolution, and DOC removal). In order to facilitate progress in the scientific community, it is imperative to move beyond this approach. It is necessary that the community forms a partnership with analytical experts in order to conduct a search for different methods that will facilitate the acquisition of new knowledge regarding the potential for difficult-to-test substances to biodegrade. One simple but powerful option is combining multiple analytical endpoints to better understand test outcomes. This was shown to be useful in screening biodegradation studies for WSPs (Menzies et al. 2023), higher tier assessments of UVCB biodegradation in river water studies (Birch et al. 2025), WSPs biodegradation during activated sludge simulation studies (Wilcox et al. 2025), and plastics biodegradation in soil (Nelson et al. 2022). The combination of the various endpoints serves to enhance the reliability of the test results obtained, as multiple endpoints are monitored concurrently. Strotmann et al. (2004) showed that the elimination of DOC invariably exceeds the generation of carbon dioxide or the depletion of oxygen. This is due to the fact that carbon is not completely oxidized; it is also incorporated into new biomass. Therefore, various endpoints can be advantageous for the estimation of heterotrophic yields, that is to say, the allocation of metabolized carbon for the purposes of energy and biomass production (Strotmann et al. 2004; Birch et al. 2025). This provides additional insight into the processes of biodegradation. On the other hand, a key disadvantage of determining multiple analytical endpoints is the significant apparatus demand of such test systems.

It is important to note that the use of specific analytical methods often provides fundamental identification of biodegradation intermediates and metabolites from assays for further evaluation. The fast development of non-target analysis methods may also in the future provide enhanced possibilities for the determination of metabolites in biodegradation tests—possibly even without the use of ^14^C-labelled substances. This is critical knowledge needed for robust biodegradation assessments and environmental risk assessments because knowledge of key metabolites from studies can inform on ecotoxicity assays if these metabolites are likely to remain present in the environmental compartment of concern for long periods of time. It is important to accurately assess biodegradation rate and extent holistically and in a robust manner before attempting to link metabolite information with ecotoxicity assays.

### Inoculum handling and characterization

As discussed in detail in the section dedicated to the characterization of inocula, the scientific community needs to embrace new methods and approaches to characterize inoculum in biodegradation assays. Many new techniques to quantify abundance and evaluate microbial communities present in biodegradation assays are available but are not being readily applied. Quantifying the actual live microbial cell counts being used at the initiation of each assay should be a minimum starting point for tests moving forward. Evaluation of the microbial community present in the inoculum and shifts in communities over the duration of the study should also be considered. Insights from this type of research could provide key information needed to develop more predictive biodegradation assays.

### Improvement of laboratory assays used to predict biodegradation

In addition to the enhancements previously outlined, it is necessary to deliberate upon the implications for biodegradation studies and the manner in which they are currently executed. Some of the key areas that need to be addressed in the development of new methods include the following: (1) methods that allow for fast cycle learning for guiding innovation of greener chemistries and assessing inoculum from a variety of different environmental locations and/or compartments; (2) methods that are designed to provide learnings on biodegradation pathways including understanding likely metabolites (if any) to help with greener design choices and ecotoxicity test design; (3) screening methods that allow for more accurate assessment of biodegradation potential possibly by increasing test duration, improving inoculum to test substance ratios, changes in inoculum treatment, and/or changing analytical endpoints for quantification; (4) methods closer to simulation study type designs that allow for prediction of biodegradation rate that can be used in quantitative risk assessment; and (5) field assays for a more accurate representation of what will happen under realistic environmental conditions.

### Weight of evidence biodegradation assessments

Biodegradation evaluations must move to fit-for-purpose testing strategies that can provide novel insights into biodegradation and be applied at different times in the innovation cycle or risk assessment schema to guide knowledge and understanding. It is essential to acknowledge and comprehend the limitations inherent to diverse assay methods and to utilize results within an appropriate context. For example, a screening enzyme study is not an appropriate assay to be used to generate information on primary and ultimate biodegradation rates that will occur under realistic environmental conditions. But instead, an enzyme assay can inform on the ability of enzymes to access and degrade a specific part of a chemical structure. In order to assess the potential for biodegradation in a holistic manner, it is also necessary to combine learnings from different models and assays, as well as read across to different chemistries. These types of integrated biodegradation evaluations would be very useful for “critical” compounds where test data may be controversial and contradictory. It would also be useful if such integrated strategies could be taken up by regulators and toolboxes with different test systems could also be standardized.

## Supplementary Information

Below is the link to the electronic supplementary material.

ESM 1 (PDF 71.5 KB)

## Data Availability

Not applicable. This is a review article based on previously published studies, and no new data were generated.
