# Supplementary material for: Testing the biodegradability of difficult compounds: a future challenge for the OECD/ISO standardization
Source: Appl Microbiol Biotechnol. 2026 Mar 24;110:122. doi: 10.1007/s00253-026-13798-x (PMC13018019; doi:10.1007/s00253-026-13798-x)
Supplement: Supplementary file 1 [file 253_2026_13798_MOESM1_ESM.pdf]

Testing the biodegradability of difficult compounds: a future challenge for the OECD/ISO standardization

Applied Microbiology and Biotechnology

Uwe Strotmann, Hermann J. Heipieper*, Christian Eberlein, Philipp Mayer, Heidi Birch, Stefan Gartiser, Udo Pagga, Soumya Daturpalli, Glauco Battagliarin, Kathleen McDonough, and Gerald Thouand

*Department of Molecular Environmental Biotechnology, Helmholtz Centre for Environmental Research - UFZ, Leipzig, Germany
e-mail: hermann.heipieper@ufz.de

Supplementary material

Reference list for Figure 1

Ahtiainen J, Aalto M, Pessala P (2003) Biodegradation of chemicals in a standardized test and in environmental conditions. Chemosphere 51(6):529-537 doi:10.1016/S0045-6535(02)00861-5

Bellia G, Tosin M, Floridi G, Degli-Innocenti F (1999) Activated vermiculite, a solid bed for testing biodegradability under composting conditions. Polym Deg Stab 66(1):65-79 doi:10.1016/S0141-3910(99)00053-1

Birch H, Andersen HR, Comber M, Mayer P (2017) Biodegradation testing of chemicals with high Henry's constants - Separating mass and effective concentration reveals higher rate constants. Chemosphere 174:716-721 doi:10.1016/j.chemosphere.2017.02.003

Birch H, Dechesne A, Sjoholm KK, Mayer P (2023) Biodegradation of chemicals tested in mixtures and individually: mixture effects on biodegradation kinetics and microbial composition. Biodegradation 34(2):139-153 doi:10.1007/s10532-022-10009-y

Birch H, Sjøholm KK, Dechesne A, Sparham C, van Egmond R, Mayer P (2022) Biodegradation kinetics of fragrances, plasticizers, UV Filters, and PAHs in a Mixture - Changing test concentrations over 5 orders of magnitude. Environ Sci Technol 56(1):293-301 doi:10.1021/acs.est.1c05583

Blok J (1994) Extrapolation of biodegradability test data by use of growth kinetic parameters. Ecotoxicol Environ Saf 27(3):306-315 doi:10.1006/eesa.1994.1024

Blok J (2000) Probability of biodegradation, a novel concept for improving chemical classification and risk assessment. Ecotoxicol Environ Saf 47(3):221-230 doi:10.1006/eesa.2000.1965

Blok J, Booy M (1984) Biodegradability test results related to quality and quantity of the inoculum. Ecotoxicol Environ Saf 8(5):410-422 doi:10.1016/0147-6513(84)90063-0

Blum JE, Stevenson CA, Stainken DM (1983) Evaluation and development of static shake-flask biodegradation test systems. Environ Toxicol Chem 2(2):141-146 doi:10.1002/etc.5620020202

Briassoulis D, Mistriotis A, Mortier N, Tosin M (2020) A horizontal test method for biodegradation in soil of bio-based and conventional plastics and lubricants. J Clean Prod 242 doi:10.1016/j.jclepro.2019.118392
Briassoulis D, Pikasi A, Papardaki NG, Mistriotis A (2020) Aerobic biodegradation of bio-based plastics in the seawater/sediment interface (sublittoral) marine environment of the coastal zone - Test method under controlled laboratory conditions. Sci Total Environ 722 doi:10.1016/j.scitotenv.2020.137748
Briassoulis D, Pikasi A, Papardaki NG, Mistriotis A (2024) Biodegradation of plastics in the pelagic environment of the coastal zone - Proposed test method under controlled laboratory conditions. Sci Total Environ 912 doi:10.1016/j.scitotenv.2023.168889
Brillet F, Cregut M, Durand MJ, Sweetlove C, Chenèble JC, L'Haridon J, Thouand G (2018) Biodegradability assessment of complex chemical mixtures using a carbon balance approach. Green Chem 20(5):1031-1041 doi:10.1039/c7gc03386a
Brown DM, Hughes CB, Spence M, Bonte M, Whale G (2018) Assessing the suitability of a manometric test system for determining the biodegradability of volatile hydrocarbons. Chemosphere 195:381-389 doi:10.1016/j.chemosphere.2017.11.169
Calmon A, Dusserre-Bresson L, Bellon-Maurel V, Feuilloley P, Silvestre F (2000) An automated test for measuring polymer biodegradation. Chemosphere 41(5):645-651 doi:10.1016/S0045-6535(99)00491-9
Calmon A, Guillaume S, Bellon-Maurel V, Feuilloley P, Silvestre F (1999) Evaluation of material biodegradability in real conditions-development of a burial test and an analysis methodology based on numerical vision. J Environ Polym Degrad 7(3):157-166 doi:10.1023/A:1022849706383
Castellani F, Esposito A, Stanzione V, Altieri R (2016) Measuring the biodegradability of plastic polymers in olive-mill waste compost with an experimental apparatus. Adv Mater Sci Eng 2016 doi:10.1155/2016/6909283
Cheng Y, Zhang K, Huang K, Zhang H (2024) Meta-Analysis and Machine Learning Models for Anaerobic Biodegradation Rates of Organic Contaminants in Sediments and Sludge. Environ Sci Technol 58(29):12976-12988 doi:10.1021/acs.est.4c01033
Chiellini E, Corti A, D'Antone S, Billingham NC (2007) Microbial biomass yield and turnover in soil biodegradation tests: carbon substrate effects. J Polym Environ15(3):169-178 doi:10.1007/s10924-007-0057-4
Chinaglia S, Tosin M, Degli-Innocenti F (2018) Biodegradation rate of biodegradable plastics at molecular level. Polym Degrad Stab 147:237-244 doi:10.1016/j.polymdegradstab.2017.12.011
da Silva SA, Faccin DJL, Cardozo NSM (2024) A Kinetic-Based Criterion for Polymer Biodegradability Applicable to Both Accelerated and Standard Long-Term Composting Biodegradation Tests. ACS Sustainable Chem Eng 12(32):11856-11865 doi:10.1021/acssuschemeng.3c03837
de Morsier A, Blok J, Gerike P, Reynolds L, Wellens H, Bontinck WJ (1987) Biodegradation tests for poorly-soluble compounds. Chemosphere 16(4):833-847 doi:10.1016/0045-6535(87)90017-8
Fischer WK, Gerike P, Holtmann W (1975) Biodegradability determinations via unspecific analyses (chemical oxygen demand, dissolved organic carbon) in coupled units of the oecd confirmatory test-I. The test. Water Res 9(12):1131-1135 doi:10.1016/0043-1354(75)90111-6

Funabashi M, Ninomiya F, Kunioka M (2007) Biodegradation of polycaprolactone powders proposed as reference test materials for international standard of biodegradation evaluation method. J Polym Environ 1515(1):7-17 doi:10.1007/s10924-006-0041-4
Gartiser S, Wallrabenstein M, Stiene G (1998) Assessment of several test methods for the determination of the anaerobic biodegradability of polymers. J Environ Polym Degrad 6(3):159-173 doi:10.1023/A:1021869530253
Gerike P (1984) The biodegradability testing of poorly water soluble compounds. Chemosphere 13(1):169-190 doi:10.1016/0045-6535(84)90018-3
Goodhead AK, Head IM, Snape JR, Davenport RJ (2014) Standard inocula preparations reduce the bacterial diversity and reliability of regulatory biodegradation test. Environ Sci Pollut Res 21(16):9511-9521 doi:10.1007/s11356-013-2064-4
Goss M, Li Z, McLachlan MS (2020) A simple field-based biodegradation test shows pH to be an inadequately controlled parameter in laboratory biodegradation testing. Environ Sci Process Impacts 22(4):1006-1013 doi:10.1039/c9em00491b
Grima S, Bellon-Maurel V, Silvestre F, Feuilloley P (2001) A new test method for determining biodegradation of plastic material under controlled aerobic conditions in a soil-simulation solid environment. J Polym Environ 9(1):39-48 doi:10.1023/A:1016044504688
Guo W, Tao J, Yang C, Song C, Geng W, Li Q, Wang Y, Kong M, Wang S (2012) Introduction of environmentally degradable parameters to evaluate the biodegradability of biodegradable polymers. PLoS One 7(5) doi:10.1371/journal.pone.0038341
Guo W, Tao J, Yang C, Zhao Q, Song C, Wang S (2010) The rapid evaluation of material biodegradability using an improved ISO 14852 method with a microbial community. Polym Test 29(7):832-839 doi:10.1016/j.polymertesting.2010.07.004
Hales SG, Philpotts CJ, Gillard C (1996) A respirometer with improved sensitivity for ready biodegradation testing. Chemosphere 33(7):1247-1259 doi:10.1016/0045-6535(96)00263-9
Handley JW, Mead C, Rausina GA, Waid LJ, Gee JC, Herron SJ (2002) The use of inert carriers in regulatory biodegradation tests of low density poorly water-soluble substances. Chemosphere 48(5):529-534 doi:10.1016/S0045-6535(02)00132-7
Herman D, Roberts DJ (2005) A marine anaerobic biodegradation test applied to the biodegradation of synthetic drilling mud base fluids. Soil Sediment Contam 14(5):433-447 doi:10.1080/15320380500180499
Huang K, Zhang H (2022) Classification and Regression Machine Learning Models for Predicting Aerobic Ready and Inherent Biodegradation of Organic Chemicals in Water. Environ Sci Technol 56(17):12755-12764 doi:10.1021/acs.est.2c01764
Ingerslev F, Nyholm N (2000) Shake-flask test for determination of biodegradation rates of 14C-labeled chemicals at low concentrations in surface water systems. Ecotoxicol Environ Saf 45(3):274-283 doi:10.1006/eesa.1999.1877
Ingerslev F, Toräng L, Nyholm N (2000) Importance of the test volume on the lag phase in biodegradation studies. Environ Toxicol Chem 19(10):2443-2447 doi:10.1002/etc.5620191008
Jayasekara R, Lonergan GT, Harding I, Bowater I, Halley P, Christie GB (2001) An automated multi-unit composting facility for biodegradability evaluations. J Chem Technol Biotechnol. 76(4):411-417 doi:10.1002/jctb.388
Jensen PD, Ge H, Batstone DJ (2011) Assessing the role of biochemical methane potential tests in determining anaerobic degradability rate and extent. Water Sci Technol 64(4):880-886

doi:10.2166/wst.2011.662
Kalsch W, Knacker T, Danneberg G, Studinger G, Franke C (1999) Biodegradation of [14C]-4-nitrophenol in a sediment-water simulation test. Int Biodeterior Biodegradation 44(1):65-74 doi:10.1016/S0964-8305(99)00061-X
Kameya T, Murayama T, Kitano M, Urano K (1995) Testing and classification methods for the biodegradabilities of organic compounds under anaerobic conditions Sci Total Environ 170(1-2):31-41 doi:10.1016/0048-9697(95)04529-A
Kijchavengkul T, Auras R, Rubino M, Ngouajio M, Thomas Fernandez R (2006) Development of an automatic laboratory-scale respirometric system to measure polymer biodegradability. Polym Test 25(8):1006-1016 doi:10.1016/j.polymertesting.2006.06.008
Kowalczyk A, Martin TJ, Price OR, Snape JR, van Egmond RA, Finnegan CJ, Schäfer H, Davenport RJ, Bending GD (2015) Refinement of biodegradation tests methodologies and the proposed utility of new microbial ecology techniques. Ecotoxicol Environ Saf 111:9-22 doi:10.1016/j.ecoenv.2014.09.021
Kuenemann P, Demorsier A, Vasseur P (1992) Interest of carbon balance in ready biodegradability testing. Chemosphere 24(1):63-69 doi:10.1016/0045-6535(92)90567-B
Kunioka M, Ninomiya F, Funabashi M (2006) Biodegradation of poly(lactic acid) powders proposed as the reference test materials for the international standard of biodegradation evaluation methods. Polym Deg Stab 91(9):1919-1928 doi:10.1016/j.polymdegradstab.2006.03.003
Larson RJ, Hansmann MA, Bookland EA (1996) Carbon dioxide recovery in ready biodegradation tests: Mass transfer and kinetic considerations. Chemosphere 33(6):1195-1210 doi:10.1016/0045-6535(96)00253-6
Lin C, Zhang H (2025) Polymer Biodegradation in Aquatic Environments: A Machine Learning Model Informed by Meta-Analysis of Structure-Biodegradation Relationships. Environ Sci Technol 59(2):1253-1263 doi:10.1021/acs.est.4c11282
Liu GJ, Frankó B, Strömberg S, Zheng D, Nistor M, Liu J, Deng LW (2023) Impact of atmospheric pressure variations on aerobic biodegradation test. Waste Manag Res 41(10):1559-1569 doi:10.1177/0734242X231164320
López-Ibáñez S, Beiras R (2022) Is a compostable plastic biodegradable in the sea? A rapid standard protocol to test mineralization in marine conditions. Sci Total Environ 831 doi:10.1016/j.scitotenv.2022.154860
Lotto NT, Calil MR, Guedes CGF, Rosa DS (2004) The effect of temperature on the biodegradation test. Mat Sci Eng C 24(5):659-662 doi:10.1016/j.msec.2004.08.009
Martin TJ, Snape JR, Bartram A, Robson A, Acharya K, Davenport RJ (2017) Environmentally Relevant Inoculum Concentrations Improve the Reliability of Persistent Assessments in Biodegradation Screening Tests. Environ Sci Technol 51(5):3065-3073 doi:10.1021/acs.est.6b05717
Martin-Aparicio A, Camenzuli L, Hughes C, Pemberton E, Saunders D, Wang NL, Lyon DY (2023) Are ready biodegradation tests effective screens for non-persistence in all environmental compartments? Environ Sci Eur 35(1) doi:10.1186/s12302-023-00769-6
Menzies J, Wilcox A, Casteel K, McDonough K (2023) Water soluble polymer biodegradation evaluation using standard and experimental methods. Sci Total Environ 858 doi:10.1016/j.scitotenv.2022.160006
Moller MT, Birch H, Sjoholm KK, Hammershoj R, Jenner K, Mayer P (2021) Biodegradation of an essential oil UVCB - Whole substance testing and constituent specific analytics yield

biodegradation kinetics of mixture constituents. Chemosphere 278 doi:10.1016/j.chemosphere.2021.130409
Montgomery HAC, Gardiner DK (1971) Experience with a bacterial inoculum for use in respirometric tests for oxygen demand. Water Res 5(4):147-163 doi:10.1016/0043-1354(71)90068-6
Norr C, Meinecke S, Brackemann H (2001) Modification of the Zahn-Wellens test: determination of the biodegradability of poorly soluble, adsorbing and volatile substances by measurement of oxygen consumption and carbon dioxide production. Chemosphere 44(4):553-559 doi:10.1016/S0045-6535(00)00497-5
Nyholm N (1990) Biodegradability testing of poorly soluble compounds by means of manometric respirometry. Chemosphere 21(12):1477-1487 doi:10.1016/0045-6535(90)90053-V
Nyholm N, Lindgaard-Jørgensen P, Hansen N (1984) Biodegradation of 4-nitrophenol in standardized aquatic degradation tests. Ecotoxicol Environ Saf 8(5):451-470 doi:10.1016/0147-6513(84)90066-6
Osswald P, Courtes R, Bauda P, Block JC, Bryers JD, Sunde E (1995) Xenobiotic biodegradation test using attached bacteria in synthetic seawater. Ecotoxicol Environ Saf 31(3):211-217 doi:10.1006/eesa.1995.1065
Ott A, Martin TJ, Whale GF, Snape JR, Rowles B, Galay-Burgos M, Davenport RJ (2019) Improving the biodegradability in seawater test (OECD 306). Sci Total Environ 666:399-404 doi:10.1016/j.scitotenv.2019.02.167
Pagga U, Beimborn DB (1993) Anaerobic biodegradation test for organic compounds.. Chemosphere 27(8):1499-1509 doi:10.1016/0045-6535(93)90244-Y
Painter HA, King EF (1983) A mathematical model of biodegradability screening tests as an aid to interpretation of observed results. Regul Toxicol Pharmacol 3(2):144-151 doi:10.1016/0273-2300(83)90039-9
Puechner P, Mueller WR, Bardtke D (1995) Assessing the biodegradation potential of polymers in screening and long-term test systems. J Environ Polym Degrad 3(3):133-143 doi:10.1007/BF02068464
Raghavan D, Wagner GC, Wool RP (1993) Aerobic biometer analysis of glucose and starch biodegradation. J. Environ Polym Degrad 1(3):203-211 doi:10.1007/BF01458028
Ress BB, Calvert PP, Pettigrew CA, Barlaz MA (1998) Testing anaerobic biodegradability of polymers in a laboratory scale simulated landfill. Environ Sci Technol 32(6):821-827 doi:10.1021/es970296h
Richterich K, Berger H, Steber J (1998) The 'two-phase closed bottle test' - A suitable method for the determination of 'ready biodegradability' of poorly soluble compounds. Chemosphere 37(2):319-326 doi:10.1016/S0045-6535(98)00049-6
Rorije E, Loonen H, Müller M, Klopman G, Peijnenburg W (1999) Evaluation and application of models for the prediction of ready biodegradability in the MITI-I test. Chemosphere 38(6):1409-1417 doi:10.1016/S0045-6535(98)00543-8
Sawada H (1998) ISO standard activities in standardization of biodegradability of plastics - development of test methods and definitions. Polym Degrad Stab 59(1-3):365-370 doi:10.1016/S0141-3910(97)00191-2
Sharabi NE, Bartha R (1993) Testing of some assumptions about biodegradability in soil as measured by carbon dioxide evolution. Appl Environ Microbiol 59(4):1201-1205 doi:10.1128/AEM.59.4.1201-1205.1993

Shrestha P, Hughes CB, Camenzuli L, Lyon D, Meisterjahn B, Hennecke T, Griffiths M, Hennecke D (2023) Improved closed test setup for biodegradation testing of slightly volatile substances in water-sediment systems (OECD 308). Chemosphere 324 doi:10.1016/j.chemosphere.2023.138294

Shrestha P, Meisterjahn B, Klein M, Mayer P, Birch H, Hughes CB, Hennecke D (2019) Biodegradation of Volatile Chemicals in Soil: Separating Volatilization and Degradation in an Improved Test Setup (OECD 307). Environ Sci Technol 53(1):20-28 doi:10.1021/acs.est.8b05079

Southwell RV, Hilton SL, Pearson JM, Hand LH, Bending GD (2020) Inclusion of seasonal variation in river system microbial communities and phototroph activity increases environmental relevance of laboratory chemical persistence tests. Sci Total Environ 733 doi:10.1016/j.scitotenv.2020.139070

Starnecker A, Menner M (1996) Assessment of biodegradability of plastics under simulated composting conditions in a laboratory test system. Int Biodeter Biodegradation 37(1-2):85-92 doi:10.1016/0964-8305(95)00089-5

Strotmann U, Reuschenbach P, Schwarz H, Pagga U (2004) Development and evaluation of an online CO_2 evolution test and a multicomponent biodegradation test system. Appl Environ Microbiol 70(8):4621-4628 doi:10.1128/AEM.70.8.4621-4628.2004

Strotmann U, Thouand G, Pagga U, Gartiser S, Heipieper HJ (2023) Toward the future of OECD/ISO biodegradability testing-new approaches and developments. Appl Microbiol Biotechnol 107(7-8):2073-2095 doi:10.1007/s00253-023-12406-6

Strotmann UJ, Eismann F, Hauth B, Bias WR (1993) An integrated test strategy for the assessment of anaerobic biodegradability of wastewaters. Chemosphere 26(12):2241-2254 doi:10.1016/0045-6535(93)90350-E

Strotmann UJ, Schwarz H, Pagga U (1995) The combined CO_2/DOC test - a new method to determine the biodegradability of organic compounds. Chemosphere 30(3):525-538 doi:10.1016/0045-6535(94)00415-Q

Struijs J, Stoltenkamp J (1990) Headspace determination of evolved carbon dioxide in a biodegradability screening test. Ecotoxicol Environ Saf 19(2):204-211 doi:10.1016/0147-6513(90)90068-G

Struijs J, Stoltenkampwouterse MJ, Dekkers ALM (1995) A rationale for the appropriate amount of inoculum in ready biodegradability tests. Biodegradation 6(4):319-327 doi:10.1007/BF00695262

Sturm RN (1973) Biodegradability of nonionic surfactants: Screening test for predicting rate and ultimate biodegradation. J Am Oil Chem Soc 50(5):159-167 doi:10.1007/BF02640470

Thouand G, Block JC (1993) The use of precultured inocula for biodegradability tests. Environ Technol 14(7):601-614 doi:10.1080/09593339309385330

Thouand G, Capdeville B, Block JC (1996) Preadapted inocula for limiting the risk of errors in biodegradability tests. Ecotoxicol Environ Saf 33(3):261-267 doi:10.1006/eesa.1996.0033

Thouand G, Friant P, Bois F, Cartier A, Maul A, Block JC (1995) Bacterial inoculum density and probability of para-nitrophenol biodegradability test response. Ecotoxicol Environ Saf 30(3):274-282 doi:10.1006/eesa.1995.1031

Van der Zee M, Stoutjesdijk JH, Feil H, Feijen J (1998) Relevance of aquatic biodegradation tests for predicting degradation of polymeric materials during biological solid waste treatment. Chemosphere 36(3):461-473 doi:10.1016/S0045-6535(97)10017-0

Van Ginkel CG, Haan A, Luuten M, Stroo CA (1995) Influence of the size and source of the inoculum on biodegradation curves in closed bottle tests. Ecotoxicol Environ Saf 31(3):218-223 doi:10.1006/eesa.1995.1066

Van Ginkel CG, Stroo CA (1992) Simple method to prolong the closed bottle test for the determination of the inherent biodegradability. Ecotoxicol Environ Saf 24(3):319-327 doi:10.1016/0147-6513(92)90008-Q

Vazquez-Rodriguez G, Goma G, Rols JL (2000) Toward a standardization of the microbial inoculum for ready biodegradability testing of chemicals. Water Sci Technol 42(5-6):43-46 doi:10.2166/wst.2000.0493

Vazquez-Rodriguez G, Goma G, Rols JL (2003) Activated sludge as inoculum for ready biodegradability testing: Effect of source. Environ Technol 24(8):979-987 doi:10.1080/09593330309385636

Vazquez-Rodriguez G, Palluy F, Goma G, Rols JL (1999) Procedures in ready biodegradability testing: Effects of the inoculation and the monitored parameter. Environ Technol 20(3):301-308 doi:10.1080/09593332008616821

Vázquez-Rodríguez GA, Beltrán-Hernández RI, Coronel-Olivares C, Rols JL (2011) Standardization of activated sludge for biodegradation tests. Anal Bioanal Chem 401(4):1127-1137 doi:10.1007/s00216-011-5212-z

Weytjens D, Vanginneken I, Painter HA (1994) The recovery of carbon dioxide in the Sturm test for ready biodegradability. Chemosphere 28(4):801-812 doi:10.1016/0045-6535(94)90232-1

Yakabe Y, Tadokoro H (1993) Assessment of biodegradability of polycaprolactone by MITI test method. Chemosphere 27(11):2169-2176 doi:10.1016/0045-6535(93)90129-S

ASTM and ISO standards mentioned in Figure 1

ASTM D5338 (2021) Standard Test Method for Determining Aerobic Biodegradation of Plastic Materials Under Controlled Composting Conditions

ASTM D5988 (2025) Standard Test Method for Determining Aerobic Biodegradation of Plastic Materials in Soil

ASTM D6691 (2024) Standard Test Method for Determining Aerobic Biodegradation of Plastic Materials in the Marine Environment by a Defined Microbial Consortium or Natural Sea Water Inoculum

ASTM D5209 (2017) Standard Test Method for Determining Aerobic Biodegradation of Plastic Materials in the Presence of Municipal Sewage Sludge

ISO 14851 (2019) Determination of the ultimate aerobic biodegradability of plastic materials in an aqueous medium — Method by measuring the oxygen demand in a closed respirometer

ISO 14852 (2021) Determination of the ultimate aerobic biodegradability of plastic materials in an aqueous medium — Method by analysis of evolved carbon dioxide.

ISO 14855 (2012 and 2018) Determination of the ultimate aerobic biodegradability of plastic materials under controlled composting conditions — Method by analysis of evolved carbon dioxide. Part 1: General method. Part 2: Gravimetric measurement of carbon

dioxide evolved in a laboratory-scale test.
ISO 17556 (2019) Plastics — Determination of the ultimate aerobic biodegradability of plastic materials in soil by measuring the oxygen demand in a respirometer or the amount of carbon dioxide evolved.
ISO 19679 (2020) Plastics — Determination of aerobic biodegradation of non-floating plastic materials in a seawater/sediment interface — Method by analysis of evolved carbon dioxide.
